# Genetic admixture and diversity in Thai domestic chickens revealed through analysis of Lao Pa Koi fighting cocks

**DOI:** 10.1371/journal.pone.0289983

**Published:** 2023-10-04

**Authors:** Pish Wattanadilokcahtkun, Piangjai Chalermwong, Worapong Singchat, Wongsathit Wongloet, Aingorn Chaiyes, Nivit Tanglertpaibul, Trifan Budi, Thitipong Panthum, Nattakan Ariyaraphong, Syed Farhan Ahmad, Artem Lisachov, Narongrit Muangmai, Mitsuo Nunome, Kyudong Han, Yoichi Matsuda, Prateep Duengkae, Kornsorn Srikulnath

**Affiliations:** 1 Faculty of Science, Animal Genomics and Bioresource Research Unit (AGB Research Unit), Kasetsart University, Bangkok, Thailand; 2 Faculty of Science, Sciences for Industry, Kasetsart University, Bangkok, Thailand; 3 Faculty of Forestry, Department of Forest Biology, Special Research Unit for Wildlife Genomics (SRUWG), Kasetsart University, Bangkok, Thailand; 4 School of Agriculture and Cooperatives, Sukhothai Thammathirat Open University, Pakkret Nonthaburi, Thailand; 5 Faculty of Science, Interdisciplinary Graduate Program in Bioscience, Kasetsart University, Bangkok, Thailand; 6 Faculty of Science, Department of Genetics, Laboratory of Animal Cytogenetics and Comparative Genomics (ACCG), Kasetsart University, Bangkok, Thailand; 7 Faculty of Fisheries, Department of Fishery Biology, Kasetsart University, Bangkok, Thailand; 8 Faculty of Science, Department of Zoology, Okayama University of Science, Okayama, Japan; 9 Department of Microbiology, Dankook University, Cheonan, Korea; 10 Bio-Medical Engineering Core Facility Research Center, Dankook University, Cheonan, Korea; 11 Center of Excellence on Agricultural Biotechnology (AG-BIO/MHESI), Kasetsart University, Bangkok, Thailand; 12 Center for Agricultural Biotechnology, Kasetsart University, Nakhon Pathom, Thailand; Ain Shams University Faculty of Agriculture, EGYPT

## Abstract

Lao Pa Koi (LPK) chicken is a popular fighting breed in Thailand, prized for (its unique characteristics acquired by selective breeding), and a valuable model for exploring the genetic diversity and admixture of red junglefowls and domestic chickens. In this study, genetic structure and diversity of LPK chicken were assessed using 28 microsatellite markers and mitochondrial DNA (mtDNA) D-loop sequences, and the findings were compared to a gene pool library from “The Siam Chicken Bioresource Project”. High genetic variability was observed in LPK chickens using mtDNA D-loop haplotype analysis, and six haplotypes were identified. Microsatellite data revealed 182 alleles, with an average of 6.5 alleles per locus. These results confirmed the occurrence of genetic admixture of red junglefowl and Thai domestic chickens in LPK chicken breed. A maximum entropy modeling approach was used to analyze the spatial suitability and to assess the adaptive evolution of LPK chickens in diverse local environments. The model identified 82.52% of the area studied as unsuitable, and 9.34%, 7.11%, and 2.02% of the area indicated moderate, low, and high suitability, respectively. The highest contribution rate to land suitability for LPK chickens was found at an elevation of 100–250 m, suggesting the importance of elevation for their potential distribution. The results of this study provide valuable insights into the genetic origin of LPK chicken breed and identify resources for future genetic improvement.

## Introduction

Domestic chickens are important in agricultural animal production as an efficient source of high-quality proteins for global food security [1–3]. Domestic chickens are considered to have been originated from a single ancestor, the red junglefowl (*Gallus gallus*, Linnaeus, 1758) [4], around 1,650–1,250 BC in Thailand [5]. Domestication involves both artificial selection of desired traits and behaviors and natural selection [5]. Highly specialized domestic chicken breeds/lines, such as layer (egg-laying) and broiler (meat-type) chickens, have been developed for specific consumption roles [6–8]. The domestication of chickens is also affected by sociocultural factors, such as their roles as entertainment, religious, and political symbols [9–11]. Globally, hundreds of phenotypically identifiable domestic chicken breeds/lines have adapted to different environmental conditions [12] and are excellent resources for future genetic improvement to prepare for climate change and food crises [13]. Historically, fighting cocks (fighting chickens) have played a significant role in cockfighting in many countries [14–16]. The Thai tradition of cockfighting has a long history and deep roots within the local culture. It involves the care, nurturing, and affection given to the birds, as well as the organization of competitive fights and the fostering of community engagement in the sport [17,18]. To gain high-quality fighting cocks with distinctive traits, selective breeding is necessary. In Thailand, high genetic admixture is common in fighting cocks for establishing high-quality fighting cocks because of selective breeding that depends on farmer preferences, for which several original fighting cock breeds, such as Lao Pa Koi, Trat, and Myanmar fighting cock, are often involved [19]. Red junglefowl or indigenous village chickens with good fighting traits were selected and bred to create original fighting cocks, and then they have been maintained within local communities [20,21]. A comparison of the genetic structure and diversity of fighting cocks with those of red junglefowl and other domestic chicken breeds would be of great significance in assisting selection breeding for accelerating genetic improvement.

Lao Pa Koi (LPK) chickens have been bred intensively in the Lamphun Province in Northern Thailand. There are approximately 100 farms in Lamphun province and neighboring provinces that breed fighting cocks for this purpose. They display distinctive phenotypic characteristics, such as a large and dominant parrot-like beak, elliptical eyes with yellow-white sclera, and a bright red-colored head comb, ears, and wattles [22]. They have a muscular body with strong and short thighs that provide robustness. Adult males weigh between 2.2–2.8 kg, whereas females are slightly smaller, weighing 1.8–2.5 kg [22]. Due to their robust physique, combative temperament, and popularity among cockfighting enthusiasts, LPK chickens with varying colors and shapes have been continuously developed by local breeders for over 40 years. They are believed to have been created by crossing a male Trat chicken (Kai Trat), a local fighting cock from Trat Province in Eastern Thailand, with an female indigenous chicken from Lamphun Province in Northern Thailand, which exhibits aggressive behavior [22]. Therefore, a genetic study of LPK chickens can offer valuable insights to clarify the process of being created a new local breed by genetic admixture between red junglefowl and domestic chickens and its impact on phenotypic diversity.

Comparing the genetic diversity and structure of LPK chickens with those of red junglefowl and other domestic chicken breeds is important for assessing their value as genetic resources and understanding the domestication process of red junglefowl. Microsatellite genotyping analysis revealed that the gene pool of most red junglefowl populations in Thailand is diverse and includes many geographically distinct populations and ecotypes in our previous studies [19,23]. The gene pool of red junglefowl differs from the ancestral populations of Thai domestic chickens, including Lueng hang khao, Chee, Pradu Hang Dam, Kheaw Paree, Betong, Decoy, fighting chickens, Nin Kaset (white), Nin Kaset (black), and Dong-Tao (Lopburi) [19,23]. LPK chicken is considered to have originated from a genetic admixture of gene pools of domestic chickens and red junglefowl especially from the Northern and Eastern ecotypes. In this study, genetic diversity of LPK chickens was examined using 28 microsatellite markers and mitochondrial DNA (mtDNA) D-loop sequences. The results were compared with a large gene pool library obtained from “The Siam Chicken Bioresource Project” (Dryad dataset: https://datadryad.org/stash/share/U8_4NYOZBF8HK4UznX4HDQl-mreFCpGZ3NNMkqhoVnc) to identify genetic footprints of red junglefowl and Thai domestic chickens in LPK chickens that have adapted to diverse local environments. Maximum entropy modeling, which enables a precise assessment of land suitability, was used to evaluate the spatial suitability of LPK chicken [24].

## 2. Materials and methods

### 2.1 Study area for land suitability of Lao Pa Koi chicken and occurrence data

**Fig 1:**
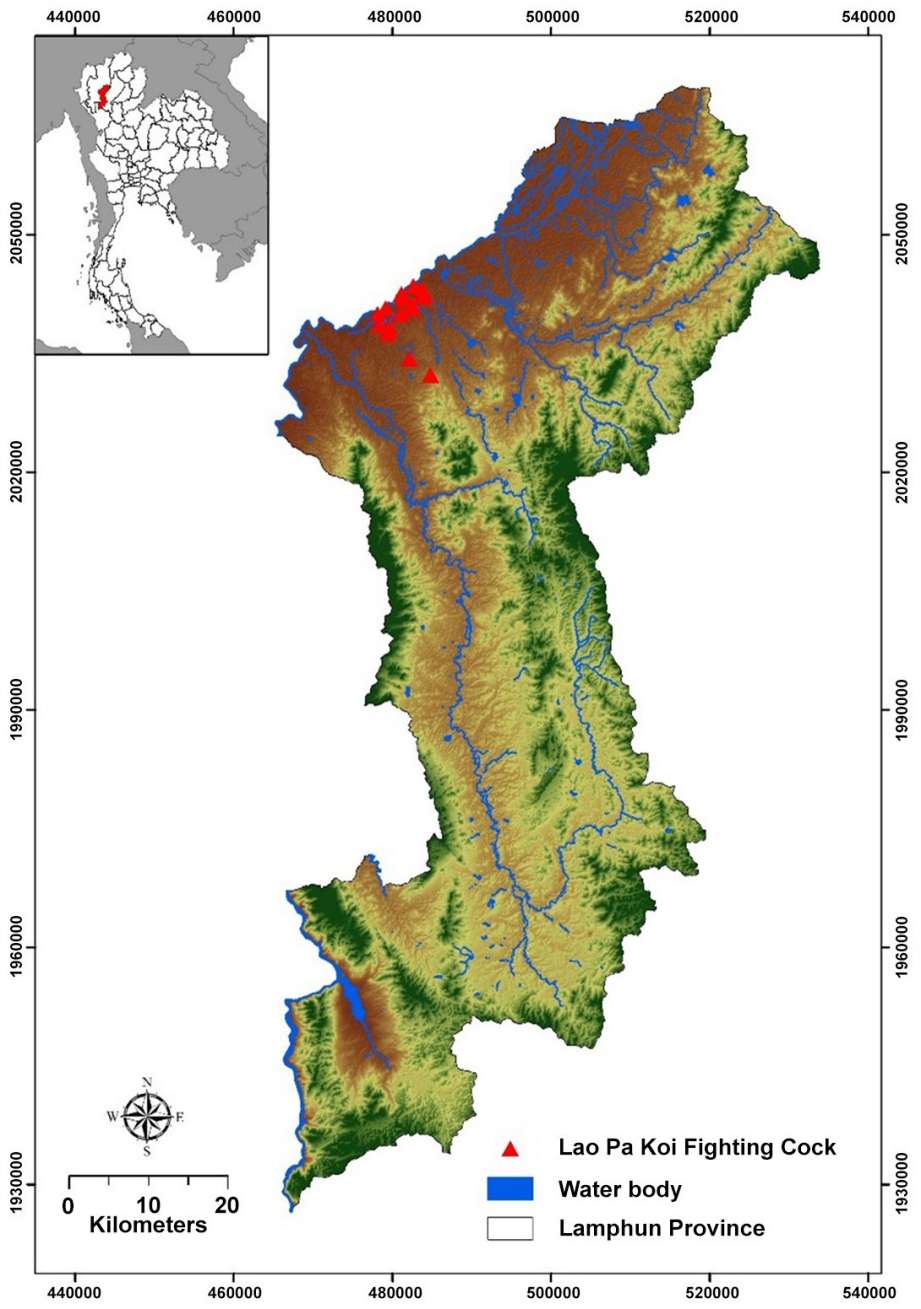
Map of studied area and locations where Lao Pa Koi chicken samples were collected.

Lamphun Province is located in the Ping River Valley in Northern Thailand (18° 34’ 49" N, 99° 0’ 26" E). The region is surrounded by mountain chains with the Thanon Thong Chai Range to the west and the Khun Tan Range to the east. With a total area of 4,478 km^2^, Lamphun is the smallest province in Northern Thailand. Data on the occurrence of LPK chickens were collected from 16 smallholder poultry and backyard chicken farms in the Pa Sang district (Fig 1), and a land suitability model for LPK chickens was constructed.

### 2.2 Environmental data

Environmental variables that potentially affect LPK chickens were included in the study, including elevation, distance to water, normalized difference vegetation index (NDVI), tree canopy cover, and forest canopy height. Altitude data (at a scale of 30-m-resolution) were obtained from the Department of National Parks, Wildlife and Plant Conservation of Thailand of the Ministry of Natural Resources and Environment (S1A Fig). The distance to water was calculated as the Euclidean distance between the main rivers (S1B Fig). Data for the inland water layers were obtained from the Land Development Department of the Ministry of Agriculture and Cooperatives of Thailand. The NDVI-estimated vegetation activity, measured using Landsat 8 satellite images between January and March 2022, was provided by the U.S. Geological Survey from the Earth Explorer website (S1C Fig). The estimated maximum tree canopy cover per pixel was obtained from Global Land Analysis and Discovery 2010 and represented as integer values (1–100%) [25] (S1D Fig). Global Landsat analysis-ready data were used to extrapolate the Global Ecosystem Dynamics Investigation footprint-level forest canopy height measurements [26] (S1E Fig), and ArcGIS was used to interpolate the environmental factors at the same spatial resolution of 30 m in a raster format.

### 2.3 Species distribution modeling

LPK chicken distribution modeling was performed using the MaxEnt algorithm in MaxEnt ver. 3.4.4 software package [24,27]. MaxEnt software utilizes location data and environmental predictors as inputs to model the distribution of LPK chickens. Location-only data have also been used in appropriate cases for species with limited distributions [28–31]. Logistic output with default settings was chosen to represent the suitability data ranging from 0 to 1, indicating the occurrence probability of LPK chickens. The convergence threshold and maximum number of iterations were set to default values (500) [27]. Regularization data were selected automatically using MaxEnt software to reduce model overfitting [30]. The optimal MaxEnt model was selected using the 10th percentile presence probability and 10-fold cross-validation method to generate a binary map. Response curves of the predictor variables were developed, and jackknife importance was tested in the final optimal model [32]. Predictive uncertainty was reduced using the ensemble forecasting approach described by Araújo and New (2007) [33], and the basic mathematical function of mean ensembles was applied to calculate the final logistic outputs following Marmion et al. (2009) [34]. Predictive performance and model validation were evaluated using receiver operating characteristics and the area under the curve (AUC) [35]. The AUC data provided a threshold-independent measure of the model accuracy, reflecting the model’s discrimination ability. An AUC value of 0 indicated no better discrimination than chance, whereas an AUC value of 1 indicated perfect discrimination. A value greater than 0.8 indicated excellent discrimination [35]. The final averaged prediction maps illustrated the presence probability of the species, with values ranging from 0 to 1. This probability was classified into five equal-sized categorical classes following Li et al. (2020) [36]: very high suitability (*p* > 0.8), high suitability (0.6 > *p* ≤ 0.8), moderate suitability (0.4 > *p* ≤ 0.6), least suitability (0.2 ≥ *p* ≤ 0.4), and no suitability (*p* < 0.2). The area under each of the classified categories was calculated using map algebra in ArcGIS.

### 2.4 Specimen collection and DNA extraction

LPK chickens were sampled from Lamphun (18°26′19′′N 98°48′55′′E); detailed information on the sampled individuals is presented in S1 Table. The Lao Pa Koi chicken, known for its excellence, has been bred in the Pa Sang district of Lamphun Province for over three decades. It is primarily distributed in this district, with limited involvement from other areas in Lamphun. The samples collected from the Pa Sang district provide valuable insights into the overall diversity of the province. The blood samples obtained from the live chickens were carefully collected from the wing vein using Vacuette^®^ 21-gauge needles. The chickens were then immediately released after blood collection. Afterwards, the collected blood was transferred into vials containing 5 mM EDTA. These vials were then stored at 4°C until they were needed for further analysis. Genomic DNA extraction was then performed using a standard salting-out protocol, as previously described [37]. DNA quality and quantity were assessed by 1% agarose gel electrophoresis and a NanoDrop 2000 Spectrophotometer (Thermo Fisher Scientific, Wilmington, DE, USA). Experimental protocols for this study were approved by the Kasetsart University Animal Experiment Committee (Approval No: ACKU65-SCI-023) and conducted in accordance with the Regulations on Animal Experiments at the Kasetsart University.

### 2.5 Mitochondrial D-loop sequencing, sequence quality control, and data analysis

Mitochondrial D-loop (mt D-loop) fragments were amplified using the primer pair Gg_D-loop_1F (5′-AGGACTACGGCTTGAAAAGC-3′) and Gg_D-loop_4R (5′-CGCAACGCAGGTGTAGTC-3′) [38]. PCR amplification and nucleotide sequencing of the DNA fragments were based on methods described by Hata et al. (2021) and Singchat et al. (2022) [19,23] (S1 File). All sequences are deposited in the DNA Data Bank of Japan (DDBJ) (https://www.ddbj.nig.ac.jp/, accessed on 22 February 2023) (accession numbers: LC761600–LC761619) (S1 Table).

### 2.6 Microsatellite genotyping and data analysis

Twenty-eight microsatellite primer sets were selected from the 30 markers recommended for chicken biodiversity studies by the Food and Agriculture Organization [39] (S2 Table). The 5′-end of the forward primer of each primer set was labeled with fluorescent dye (6-FAM or HEX; Macrogen Inc., Seoul, Korea). PCR amplification for microsatellite genotyping and analysis of genetic diversity and population structure based on microsatellite data were performed as described previously [19,23] (S1 File). Genotypic data generated in this study are deposited in the Dryad Digital Repository Dataset of The Siam Chicken Bioresource Project (https://datadryad.org/stash/share/U8_4NYOZBF8HK4UznX4HDQl-mreFCpGZ3NNMkqhoVnc, accessed on 10 February 2023).

## 3. Results

### 3.1. Land suitability of Lao Pa Koi chickens

**Fig 2:**
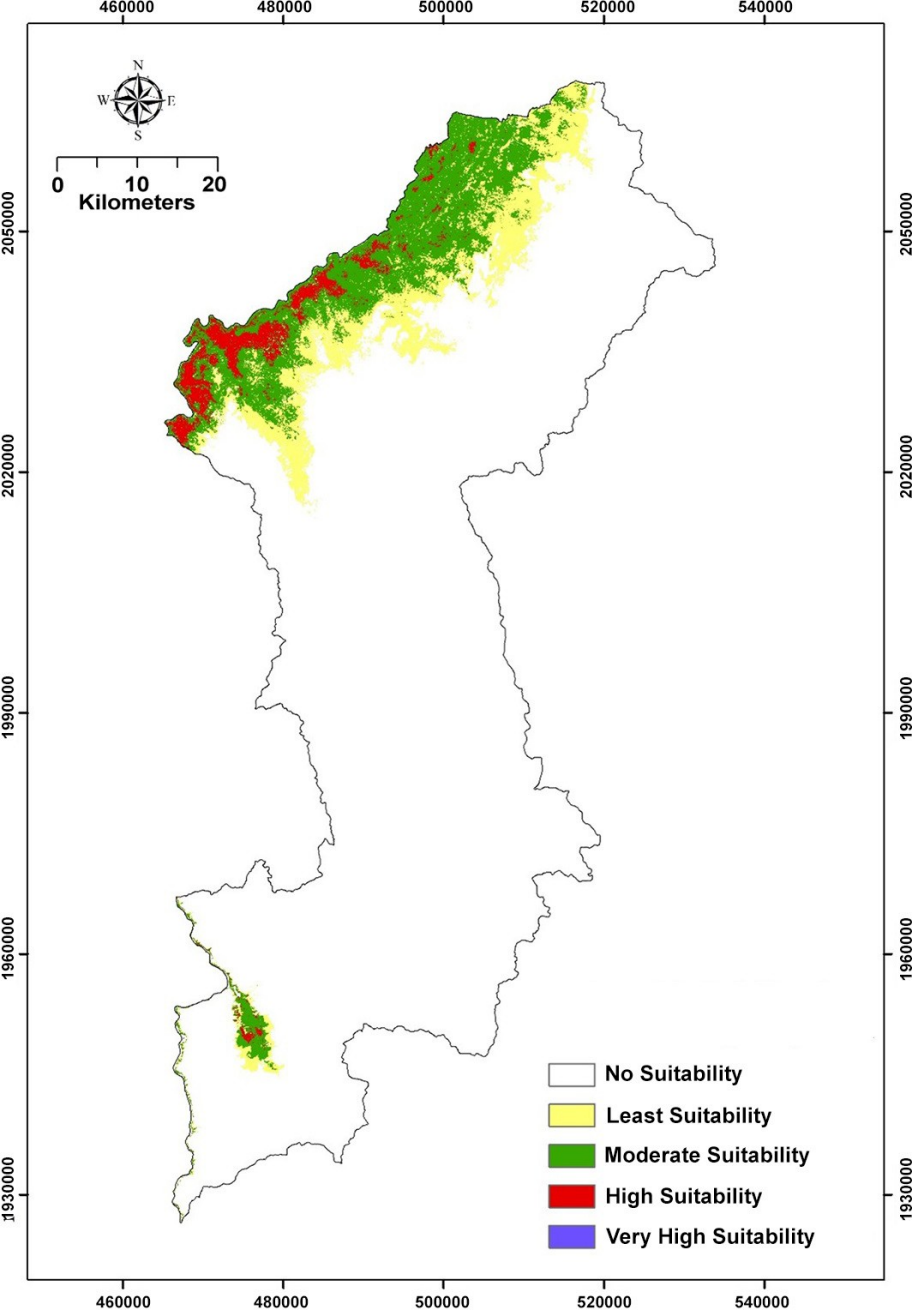
Potential land suitability for Lao Pa Koi chickens in Lamphun Province.

Lamphun Province has an area of 11,460 km^2^. The area of land unsuitable for the inhabitation of LPK chickens was estimated to be 3,651 km^2^ (81.53%) by the prediction model, followed by that for moderate, low, and high suitability to be 418 km^2^ (9.34%), 318 km^2^ (7.10%), and 91 km^2^ (2.03%) (Fig 2). Marginal response curves were applied to show the impact of environmental variations on the occurrence probability. Optimal environmental conditions, including in elevation (100–250 m), tree canopy cover (0–5%), forest canopy height (0–5 m; shrub), distance to the main river (14,000 m), and NDVI (−0.15–0.50); area of built up, fallow, crop, grass and agroforestry), are presented in S2 Fig.

### 3.2. Model performance and variable importance

The Maxent model was effective in predicting the potential distribution of LPK chickens, as indicated by the very high training AUC value of 0.921 from the average model (10 replication runs). The Jackknife method was applied to the Maxent model, and the results showed a weighting effect of different environmental factors on land suitability for LPK chickens (S3 Fig). The potential distribution of LPK chickens was affected by environmental factors, such as elevation, tree canopy cover, forest canopy height, distance to water, and NDVI, with contribution rates of 72.0%, 22.9%, 3.2%, 1.8%, and 0.1%, respectively.

### 3.3. Genetic variability among LPK chickens based on mitochondrial DNA haplotype analysis

**Fig 3:**
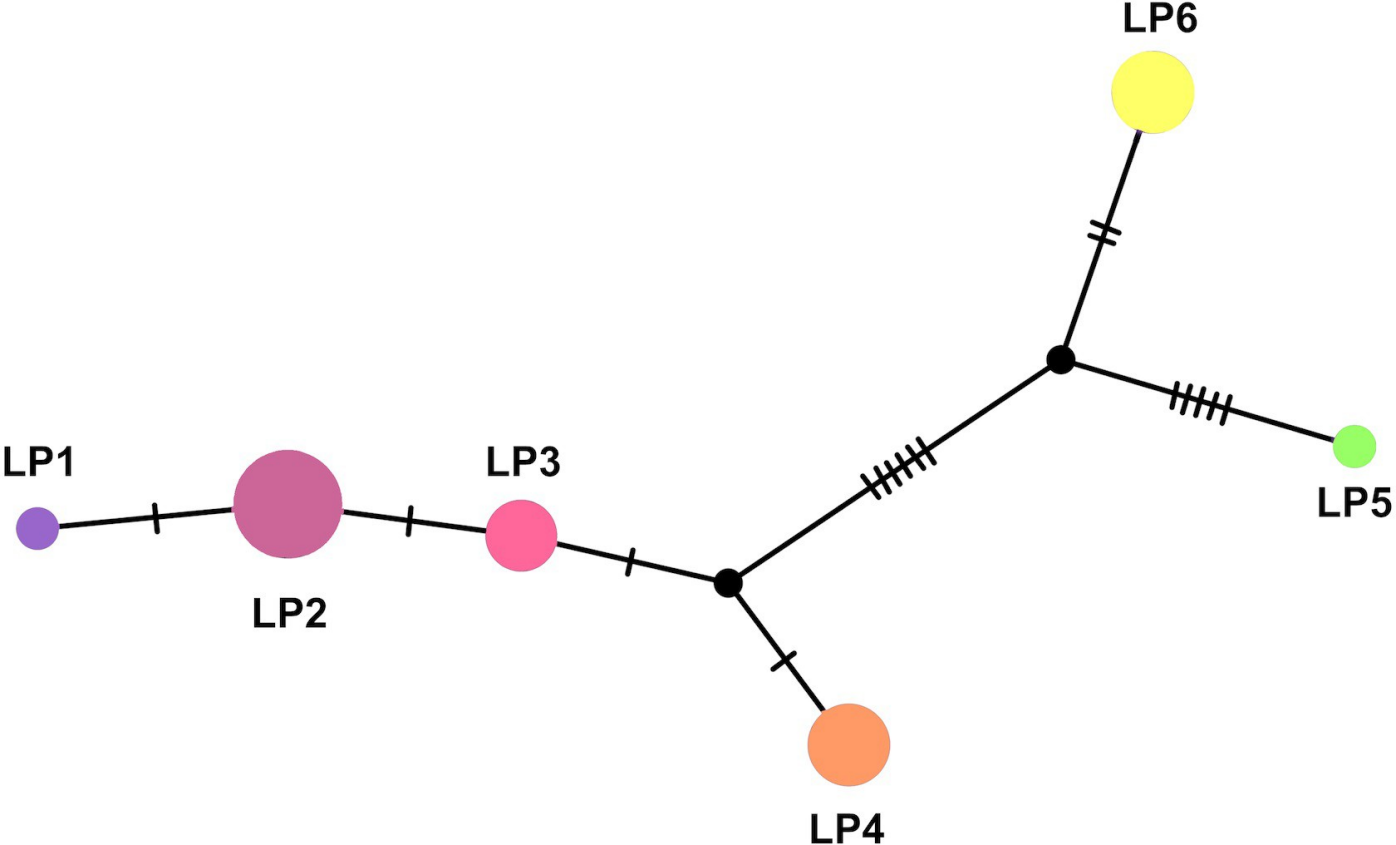
Haplotype network based on the sequence data of the mitochondrial DNA D-loop region in Lao Pa Koi chickens derived from (the Lamphun farmer community), Lamphun, Thailand.

The amplicon and alignment lengths of the mtDNA D-loop sequences of the six haplotypes from LPK chickens were 1,200 bp and 700–850 bp, respectively. Haplotype and nucleotide diversities were 0.811 ± 0.052 and 0.007 ± 0.004, respectively. Theta (per site) from *S* and average number of nucleotide differences (*k*) were 0.007 and 5.005, respectively. A complex haplotype network and phylogenetic tree were constructed using a large number of polymorphic sites and haplotypes. Haplotype B, represented by LP2, was the most frequent in LPK chickens, whereas the other haplotypes were assigned to haplogroups CD and F (Fig 3). To examine genetic differentiation among domestic chicken breeds and red junglefowl populations, Wright’s F-statistic was calculated for subpopulations within each population (*F*_ST_). Genetic differentiation was significant (*p* < 0.05) between most populations and breed pairs (S3 Table).

### 3.4. Genetic variability among Lao Pa Koi chickens based on microsatellite data

**Fig 4:**
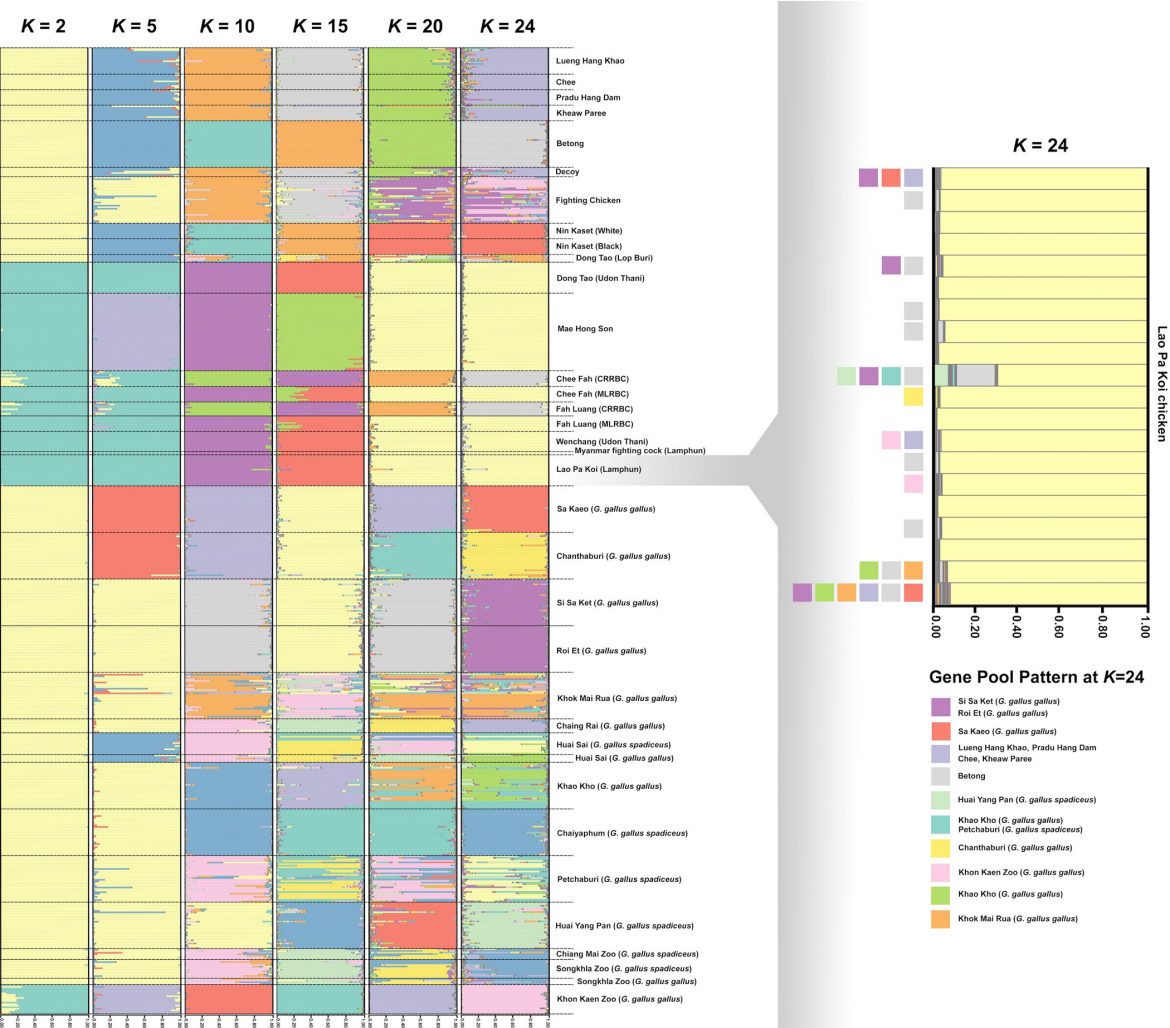
Population genetic structures of Lao Pa Koi chickens, red junglefowl, and domestic chicken breeds in Thailand. Each vertical bar on the x-axis represents an individual chicken; the y-axis represents the posterior probability in each population.

**Fig 5:**
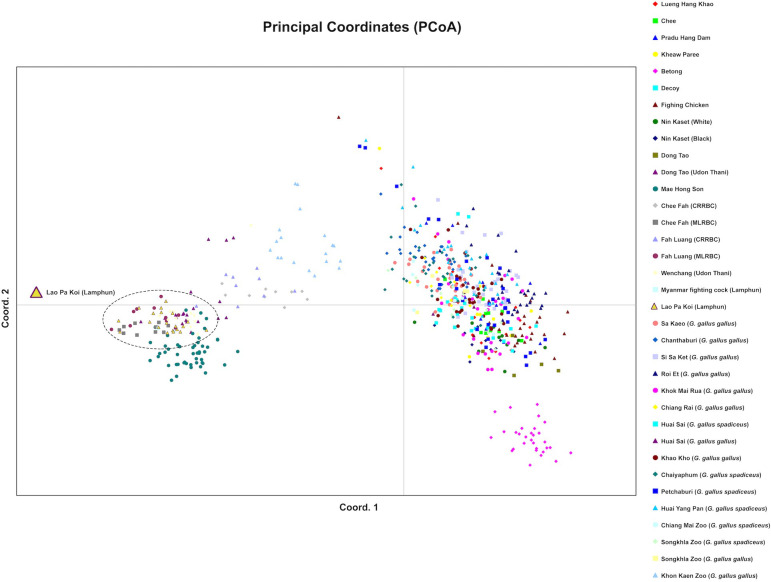
Principal component analysis of Lao Pa Koi chickens, Thailand, with red junglefowl and domestic chicken breeds in Thailand.

**Fig 6:**
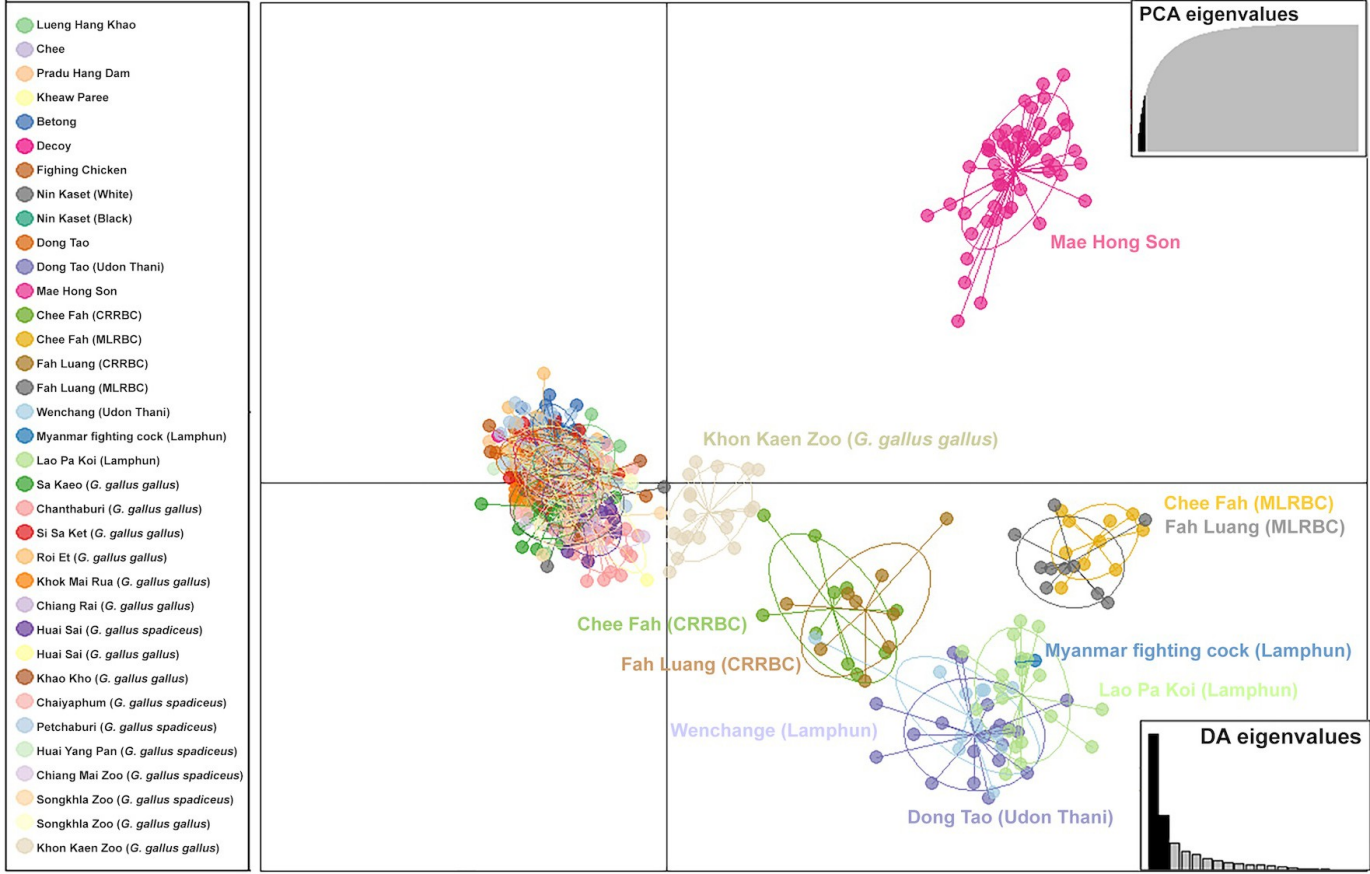
Discriminant Analysis of Principal Components (DAPC) of Lao Pa Koi chickens with red junglefowl and other domestic breeds. Scatter plots based on the DAPC output for three assigned genetic clusters are indicated by different colors. The dots represent each individual.

A total of 182 alleles were detected (n = 20) in LPK chickens, with a mean number of 6.5 ± 0.536 alleles per locus (Table 1). Significant departures from Hardy-Weinberg expectations were observed at 7 loci, with evidence of linkage disequilibrium (S4 Table). However, due to small sample sizes, the ability to detect significant departures from Hardy-Weinberg equilibrium was limited. No consistent patterns of deviation from Hardy-Weinberg equilibrium or linkage equilibrium were detected across the sites. Therefore, all microsatellite loci were used for the genetic analyses. All markers were analyzed similarly because no null alleles were detected. LPK chickens exhibited a negative *F* statistic (*F*-value). The polymorphic information content (*PIC*) was 0.620 ± 0.029, whereas the Shannon’s information index (*I*) was 1.383 ± 0.083 (S5 Table). The *H*_o_ and *H*_e_ values were 0.744 ± 0.042 and 0.662 ± 0.028 (mean ± standard error [SE]), respectively (Tables 1 and S5), which were significantly different from each other by Welch’s t-test (*t* = 7.265, df = 0.082, *p* < 0.01). The *AR* value of the population was 6.411 ± 0.524, with genetic diversity indices that are summarized in Tables 1 and S5. A pairwise test was performed to determine the level of relatedness (*r*) among individual LPK chickens in the population. Mean pairwise *r* value of 190 combination pairs among 20 LPK chickens was –0.025 ± 0.002, with all pairs displaying –0.25 < *r* < 0.25 (Tables 2 and S6). The mean *F*_IS_ value was –0.145 ± 0.023 in LPK (Table 2), ranging from −0.184 to −0.112 (S7 Table). Genetic differentiation was mostly observed between populations/breeds with significant differences (*p* < 0.05) in *F*_ST_ values (S8 Table). The gene pool of LPK chickens was compared with the baseline reference data from our previous studies, including red junglefowls and Thai domestic chicken breeds [17,21]. Various population structure patterns were revealed using the Bayesian model-based clustering algorithm implemented in STRUCTURE, with *K*-values ranging from 2 to 25 (Fig 4). The highest posterior probability in STRUCTURE analysis based on Evanno’s Δ*K* [40] was found with one peak at *K* = 2, while based on the mean ln P(*K*), the one peak was observed at *K* = 25 (S4 Fig). Diverse pattern of gene pool was observed for red junglefowl in contrast to a uniform gene pools that were observed in Lueng Hang Khao, Chee, Pradu Hang Dam, Kheaw Paree, Betong, Nin Kaset (White), Nin Kaset (Black), Dong Tao (Udon Thani), Mae Hong Son, Chee Fah (CRRBC), Chee Fah (MLRBC), Fah Luang (CRRBC), Fah Luang (MLRBC), Wenchang (Udon Thani), Myanmar fighting cock (Lamphun), and LPK (Lamphun). Gene pool patterns of Lueng Hang Khao, Chee, Pradu Hang Dam or Keaw Paree breeds were similar to each other; however, a part of the gene pool of red junglefowl, derived from Si Sa Ket (*G*. *gallus gallus*), Roi Et (*G*. *gallus gallus*), Khon Kaen Zoo (*G*. *gallus gallus*), Huai Sai (*G*. *gallus spadiceus*), Sa Kaeo (*G*. *gallus gallus*), Chiang Rai (*G*. *gallus gallus*), Huai Yang Pan (*G*. *gallus spadiceus*), Khok Mai Rua (*G*. *gallus gallus*), Khao Kho (*G*. *gallus gallus*), Petchaburi (*G*. *gallus spadiceus*), and Chanthaburi (*G*. *gallus gallus*) was identified in the gene pool of LPK chickens at *K* = 24. The gene pool of LPK chickens was closely related to those of domestic chickens and red junglefowl derived mainly from Huai Sai (*G*. *gallus spadiceus*) and Petchaburi (*G*. *gallus spadiceus*), with no detectable genetic selective sweeps in any chicken breeds (S5 Fig). Multiple clusters were observed in the PCoA and DAPC analyses (Figs 5 and 6), with the major cluster containing several red junglefowl populations and domestic chicken breeds. LPK chickens were grouped separately into Mae Hong Son chickens, Chee Fah and Fah Luang chickens derived from Mae Hong Son, and Dong Tao chickens derived from Udon Thani, Wenchang chickens, and Myanmar fighting cock.

**Table 1:** Genetic diversity of Lao Pa Koi chickens (n = 20) based on 28 microsatellite loci.

Population		*N*_a_ ^1^	*AR* ^2^	*N*_ea_ ^3^	*I* ^4^	*H*_o_ ^5^	*H*_e_ ^6^	*PIC* ^7^	*F* ^8^
Lamphun	Mean	6.500	6.411	3.561	1.383	0.744	0.662	0.620	−0.102
	S.E.	0.536	0.524	0.325	0.083	0.042	0.028	0.029	0.036

^1^Number of alleles, *N*_a_

^2^Allelic richness, *AR*

^3^Number of effective alleles, *N*_ea_

^4^Shannon’s information index, *I*

^5^Observed heterozygosity, *H*_o_

^6^Expected heterozygosity, *H*_e_

^7^Polymorphic information content, *PIC*

^8^Fixation index, *F*.

**Table 2:** Inbreeding coefficient, relatedness, effective population size, ratio of effective population size, and confidence interval in Lao Pa Koi chickens derived from the Numdib subdistrict and Pasang district, Lamphun. The estimates were calculated using NeEstimator version 2.1 [41], COANCESTRY version 1.0.1.9 [42], and GenAlEx version 6.5 [43]. Detailed information on each Lao Pa Koi chicken is presented in S6 and S7 Tables.

Population	N^1^	*F*_IS_ ^2^	Relatedness (*r*)	Estimated *N*_e_^3^	95% CIs for *N*_e_^4^	*N*_e_*/*N
Lamphun	20	−0.145±0.023	−0.025±0.002	183	70.8–81.5	9.15

^1^Sample size, N

^2^Inbreeding coefficient, *F*_IS_

^3^Effective population size, *N*_e_

^4^Confidence interval, CI.

## 4. Discussion

Domestic chickens are now used for various purposes owing to their potential, such as food source, colorful appearance, characteristic songs, and gamecock [44–48]. Fighting cocks were historically used for ceremonial and political events in the Kingdom of Thailand (Siam) and other Southeast Asian kingdoms, as seen during the reigns of King Ram Khamhaeng the Great (1278–1298) and King Naresuan the Great (1590–1605) [49]. In this study, the genetic analyses were conducted focusing on the assessment of genetic diversity and structure of Lao Pa Koi fighting cock population and comparison of the result with our current collection data of red junglefowl and various domestic chicken breeds. Red junglefowl and indigenous village chickens are probably free-ranging scavengers, and no significant differences were found between *H*_o_ and *H*_e_ in their populations as described in the Siam Chicken Bioresource Project library [19,23]. By contrast, a remarkable difference between *H*_o_ and *H*_e_ was observed for LPK chicken population, where *H*_o_ was higher than *H*_e_, and there was no subpopulation within LPK chicken population examined (negative *F* value). This higher heterozygosity than expected may reflect that genetic contamination, such as outbreeding with individuals from populations with different origins or ecotypes, occurred during the process of breeding in the past [50,51]. In the Lamphun region, a suitable environment for both LPK chickens and red junglefowl is provided by the forest. The highest contribution rate to land suitability for LPK chickens was found at an elevation range of 100–250 m, providing the potential distribution that may cause the hybridization crossing between LPK chickens and red junglefowl.

### 4.1. Lao Pa Koi chicken shares different ecotypes of red junglefowl

Fighting cocks were often free-ranging during domestication, resulting in frequent genetic admixture between domesticated chickens and red junglefowl [19,52,53]. Our microsatellite data revealed that LPK chickens shared a partial gene pool with red junglefowl, particularly from East and lower North ecotypes. The majority of mtDNA D-loop haplotypes of LPK chickens belonged to haplogroups B and CD, which were also dominant in red junglefowl in Thailand. This suggests that LPK chicken may have been established by a genetic admixture between North and East ecotypes of red junglefowls and potential indigenous chickens (see gene pool pattern in Fig 4, *K* = 24). The gene pool of North-East or South ecotypes of red junglefowl was also detected in LPK chickens, which is likely attributed to the widespread distribution of red junglefowls throughout Thailand [23]. It is therefore assumed that genetic differences may exist between LPK chicken and other common fighting cocks that was established by selective breeding such as artificial crossbreeding between multiple breeds/lines [54,55]. However, Trat chicken specimens could not be collected in Thailand to compare their data with that of LPK chicken because the pure Trat chicken breed is nearly extinct. A small portion of LPK chicken gene pool was shared with ornamental indigenous chicken breeds, such as Luang Hang Khao, Chee, Pradu Hang Dam, and Kheaw Paree, which have undergone intensive human-mediated selection. One individual among LPK chickens was found to contain the mtDNA D-loop of haplogroup F. Hata et al. (2021) [19] reported that haplogroup F was only observed in Luang Hang Khao and Pradu Hang Dam indigenous chicken breeds, but not found in red junglefowl populations from Thailand. This also suggests the presence of genetic footprint from other domestic chicken breeds in LPK chickens. The distribution of haplotype F is limited to Yunnan Province in China, Thailand, and Myanmar [56,57], indicating the potential influence of Chinese and Myanmar indigenous chickens on the selective breeding of Thai indigenous chickens. Further investigation is needed to determine whether there is any significant genetic admixture or introgression from exotic chicken breeds or red junglefowl from outside of Thailand; however, the present result suggests that such an admixture is limited and unnoticeable. Interestingly, LPK chicken, Mae Hong Son, Chee Fah, and Fah Luang chickens from Mae Hong Son Province exhibited similar partial gene pool patterns at various *K* values, whereas the gene pools of Luang Hang Khao, Chee, Pradu Hang Dam, and Kheaw Paree chicken breeds were different from them. These differences in gene pools of domestic chicken breeds may reflect the extent of selection breeding.

### 4.2. Gene pool of Thai domestic chickens

Since domestication of chickens occurred around 8000 years ago, intensive crossbreeding among genetically divergent chickens, including occasional cases of genetic introgression into indigenous chickens from red junglefowl, has been globally practiced to establish the chicken breeds that meet to a variety of social needs [5,58]. A variety of phenotypes of comb shape, skin color, and feather color as observed in Pradu Hang Dam, Lueng Hang Khao, Kheaw Paree, and Chee chickens (ornamental domestic chickens) have been preferred and selected by smallholder farmers [19,59,60]. Pradu Hang Dam, Lueng Hang Khao, Kheaw Paree, and Chee chickens were classified into the same gene pool clusters across different *K* values. Evidence of a slight genetic admixture was found in these chicken breeds, as the *K* values increased progressively in the STRUCTURE plot compared to the Decoy chicken. This suggests that the genomic landscapes of these chicken breeds have been formed by long-term selective breeding. The Decoy chicken, which is used to capture red junglefowl from the wild, may not have undergone long-term selective breeding. By contrast, common fighting chickens show a large genetic admixture due to intercrossing of various breeds/lines for improving their physical features, such as comb shape, height, weight, shank length, muscle strength, and offensive ability required for fighting [19,23,61]. The frequent exchange of individuals allows the spread of diverse alleles among common fighting chickens throughout the country [62]. Compared to other domestic chickens such as Chee Fah, Faf Luang, Mae Hong Son, Pradu Hang Dam, and Kheaw Paree, lower heterogeneity in LPK chickens with high *F*_ST_ values based on both microsatellite genotypes and mtDNA D-loop sequences may have resulted from mating restricted within the breed. LPK, Chee Fah, Far Luang, and Mae Hong Son chickens may share gene pools because they were derived from the same ancestral origin; however, microsatellite genotyping provides only a limited resolution in distinguishing between these breeds.

**Fig 7:**
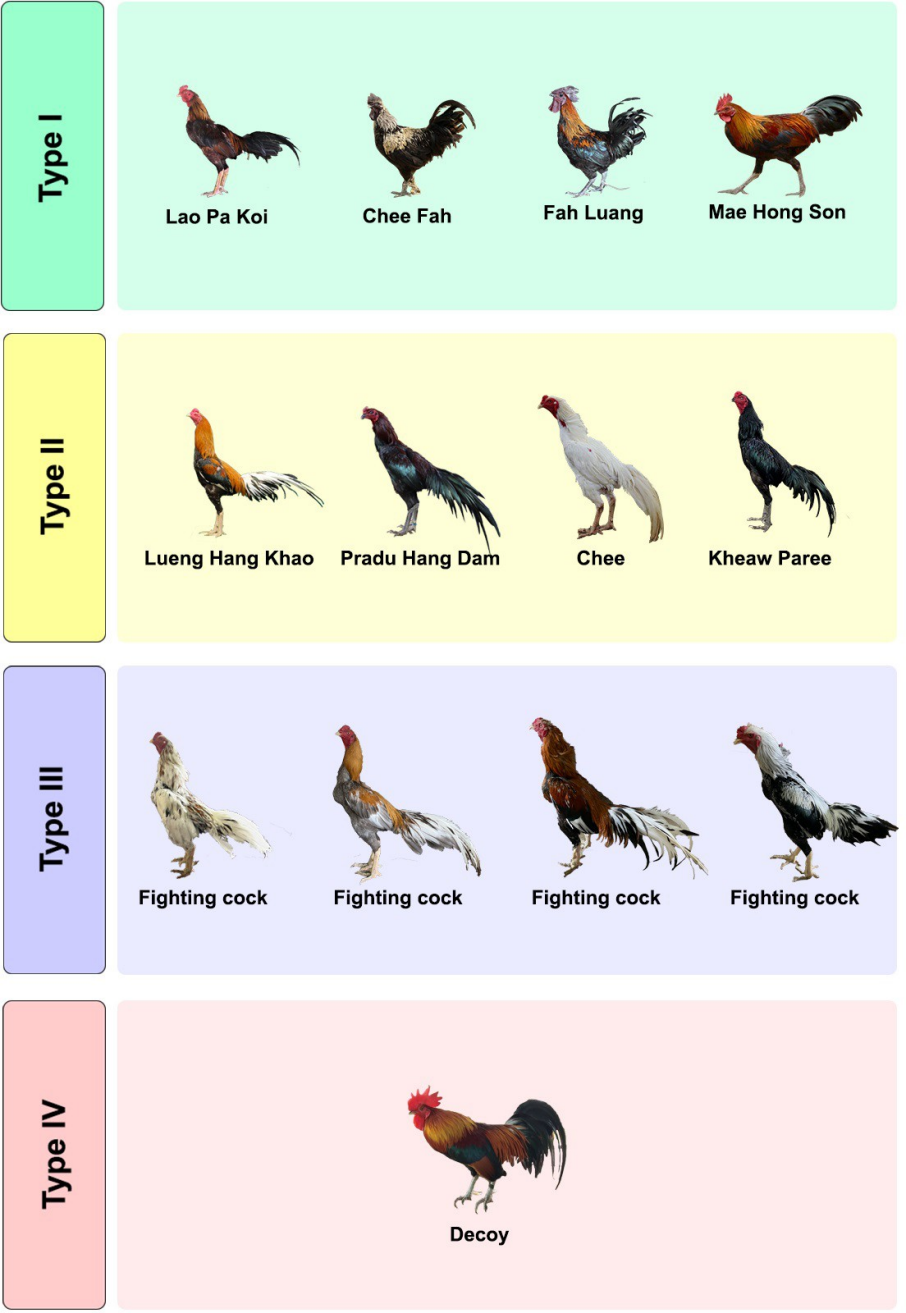
Types of Thai domestic chickens based on the difference of gene pool patterns.

Thai domestic chickens can be mainly classified into four types based on their gene pool structures. Type I includes indigenous chicken breeds or local fighting cocks, such as LPK, Betong, Chee Fah, Fah Luang, Wenchang, Myanmar Fighting Chicken, Dong Tao and Mae Hong Son chickens, which have been domesticated and persisted in agricultural societies or by local fanciers of cock fight for decades or even centuries (Fig 7). The adaptation to local environmental conditions in LPK chicken may have resulted from a long history of genetic changes via natural selection and/or genetic drift in this breed. Type II breeds that have been selected for a wide variety of desired morphological traits, such as Pradu Hang Dam, Lueng Hang Khao, Kheaw Paree, and Chee, have been maintained by fanciers. A wide variety of genetic characteristics in these breeds may have been acquired by human-mediated selection that was rapidly implemented in relatively small populations [63]. Type III chicken breeds, known as the primary commercial fighting cocks, have been artificially selected for desirable traits required for cockfighting, such as strength, aggression, and pain tolerance. The Decoy chicken is classified as type IV, that is, a local domesticated breed that has not had selective breeding. The gene pool of Decoy chickens had a genetic contribution from red junglefowl after domestication [19]. No commercial chicken breeds (layer and meat types) are included in this classification because they are international breeds that have a long history of selective breeding. Genotyping with DNA markers, phenotypic characterization, and breeding histories of populations can aid in selecting populations that meet the practical use of food production and conservation of resources. However, limited sources of population histories and genetic drift may make it difficult to prove the history of selective sweeps in several chicken breeds just by using microsatellite markers. Therefore, genome-wide analysis of single nucleotide polymorphisms (SNPs) and whole-genome sequencing (WGS) with larger sample sizes will be required for a more detailed and comprehensive investigation in the future. To record the characteristics of each breed, identification of genetic markers associated with phenotypic traits is required. Indigenous and local chickens have substantial genetic diversity, which provide a reservoir of potentially useful genetic traits, such as the adaptability to a variety of environmental conditions, for contributing to the improvement of chicken breeds.

## 5. Conclusions

This study revealed a complex landscape of genetic diversity of LPK fighting cock, which was caused by the genetic admixture between domestic chickens and red junglefowl that occurred during the process of breeding of LPK chickens. According to their historical records of breeding and sampling sites, and comparison with the genetic data of other domestic chicken populations in Thailand, it was revealed that the northern and eastern ecotypes of red junglefowl contribute to the gene pool of LPK chickens by natural selection and artificial selection dedicated to cockfighting. To provide future management recommendations, a higher molecular-level diversity assessment is required for domestic lines, even in fighting cocks.

## Supporting information

S1 Fig Environmental variables used to assess the species distribution model of Lao Pa Koi chickens: (a) Elevation, (b) Distance to river, (c) Normalized difference vegetation index (NDVI), (d) Tree canopy cover, and (e) Forest canopy height.
(TIFF)

S2 Fig Response area curves illustrating the relationship of MaxEnt predicted probability of occurrence to environmentao variables influencing the distribution of local Lao Pa Koi chickens. (a) Elevation, (b) Tree canopy cover (b) Forest canopy height, (d) Distance to main river, and (e) Normalized difference vegetation index (NDVI).
(TIFF)

S3 Fig MaxEnt model showing the Jackknife test of the importance of environmental variables affecting the potential distribution of Lao Pa Koi chickens. (TIFF)

S4 Fig Genetic population structures of the Siam Chicken Bioresource Project and Lao Pa Koi chickens (n = 20) generated using the Bayesian model-based clustering algorithms implemented in STRUCTURE. Delta *K* and L(*K*) values were calculated using STRUCTURE version 2.3.4 that was parallel run using *Structure_threader*. (a) Plot of Evanno’s Δ*K*. (b) Plot of ln P(*K*).
(TIFF)

S5 Fig Mapping of expected heterozygosity (*H*_e_) against inbreeding coefficients (*F*_IS_) along the length of the physical map. (a) Lao Pa Koi populations. (b) microsatellite loci.
(TIFF)

S1 Table Specimens collected from 16 smallholder poultry and backyard chicken farms in the Pa Sang district of Lao Pa Koi chickens in Thailand. All sequences (accession number LC761600–LC761619) are deposited in the DNA Data Bank of Japan (DDBJ).
(DOCX)

S2 Table Microsatellite primers and their nucleotide sequences used for the present study. (DOCX)

S3 Table Genetic differentiation between Lao Pa Koi chickens (the present study) and other domestic chickens reported in our previous studies (Hata et al., 2021 [19], Singchat et al., 2022) [23], based on the mitochondrial D-loop sequence. FST, Wright’s F-statistics for subpopulations within the total population.
(DOCX)

S4 Table Pairwise comparison of linkage disequilibrium of 28 microsatellite loci in Lao Pa Koi chickens. Numbers indicate *p*-values with 110 permutations.
(DOCX)

S5 Table Genetic diversity of Lao Pa Koi chickens based on 28 microsatellite loci. (DOCX)

S6 Table Pairwise genetic relatedness (*r*) among Lao Pa Koi chickens. (DOCX)

S7 Table Inbreeding coefficients (*F*_IS_) of each individual of Lao Pa Koi chickens. (DOCX)

S8 Table Genetic differentiation between Lao Pa Koi chickens (the present study) and other domestic chickens reported in our previous studies and the Siam Chicken Bioresource Project (Hata et al., 2021 [19], Singchat et al., 2022 [23]), based on 28 microsatellite loci. *F*_ST_, Wright’s F-statistics for subpopulations within the total population.
(DOCX)

S9 Table List of indigenous chicken breeds and red junglefowl populations examined in the present study. (DOCX)

S1 File Supplementary material. (DOCX)
