# Supplementary figures and images for: Genetic admixture and diversity in Thai domestic chickens revealed through analysis of Lao Pa Koi fighting cocks
Source: PLoS One. 2023 Oct 4;18(10):e0289983. doi: 10.1371/journal.pone.0289983 (PMC10550135; doi:10.1371/journal.pone.0289983)

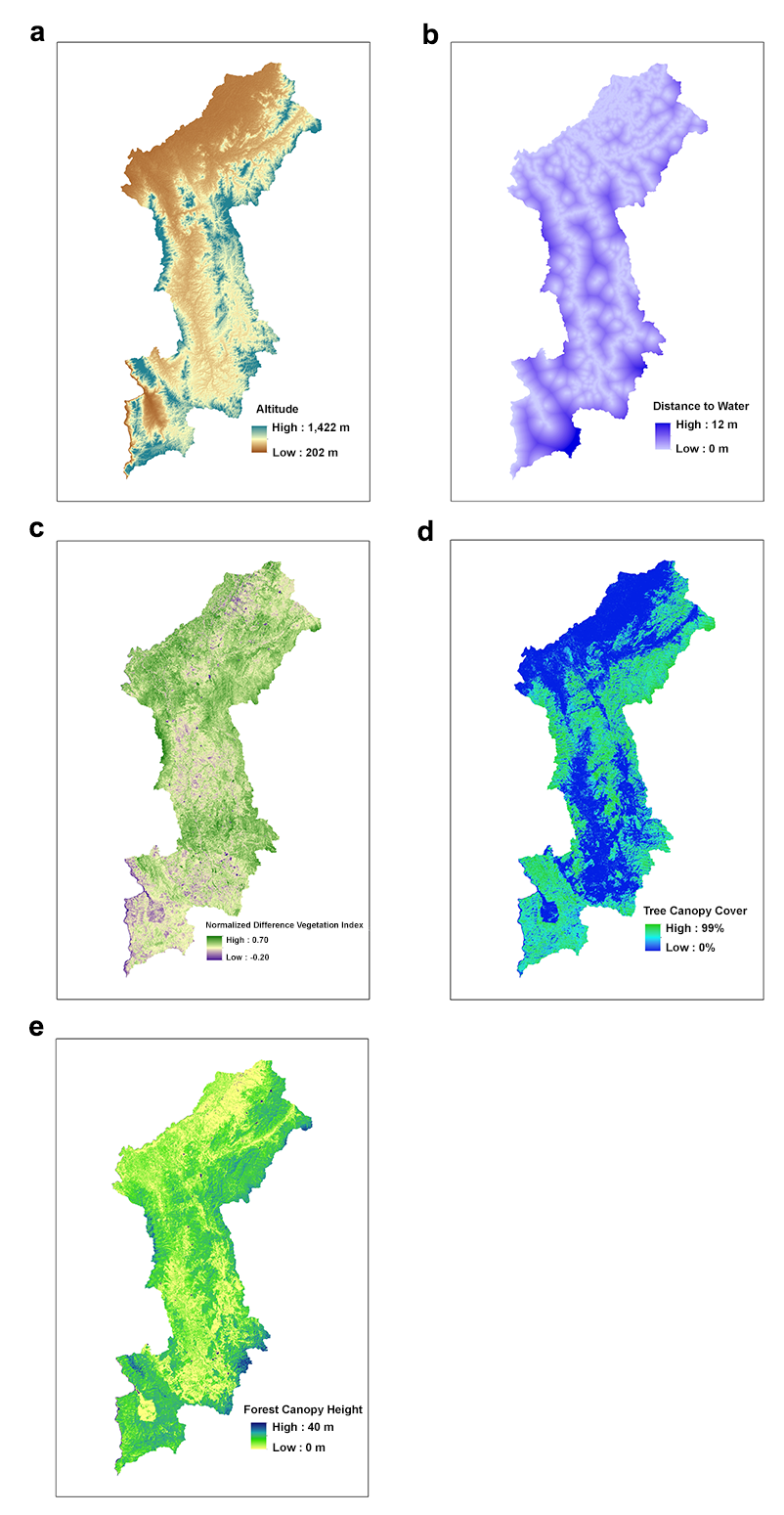

Supplement: S1 Fig — Environmental variables used to assess the species distribution model of Lao Pa Koi chickens: (a) Elevation, (b) Distance to river, (c) Normalized difference vegetation index (NDVI), (d) Tree canopy cover, and (e) Forest canopy height. (TIFF) [file pone.0289983.s001.tiff]

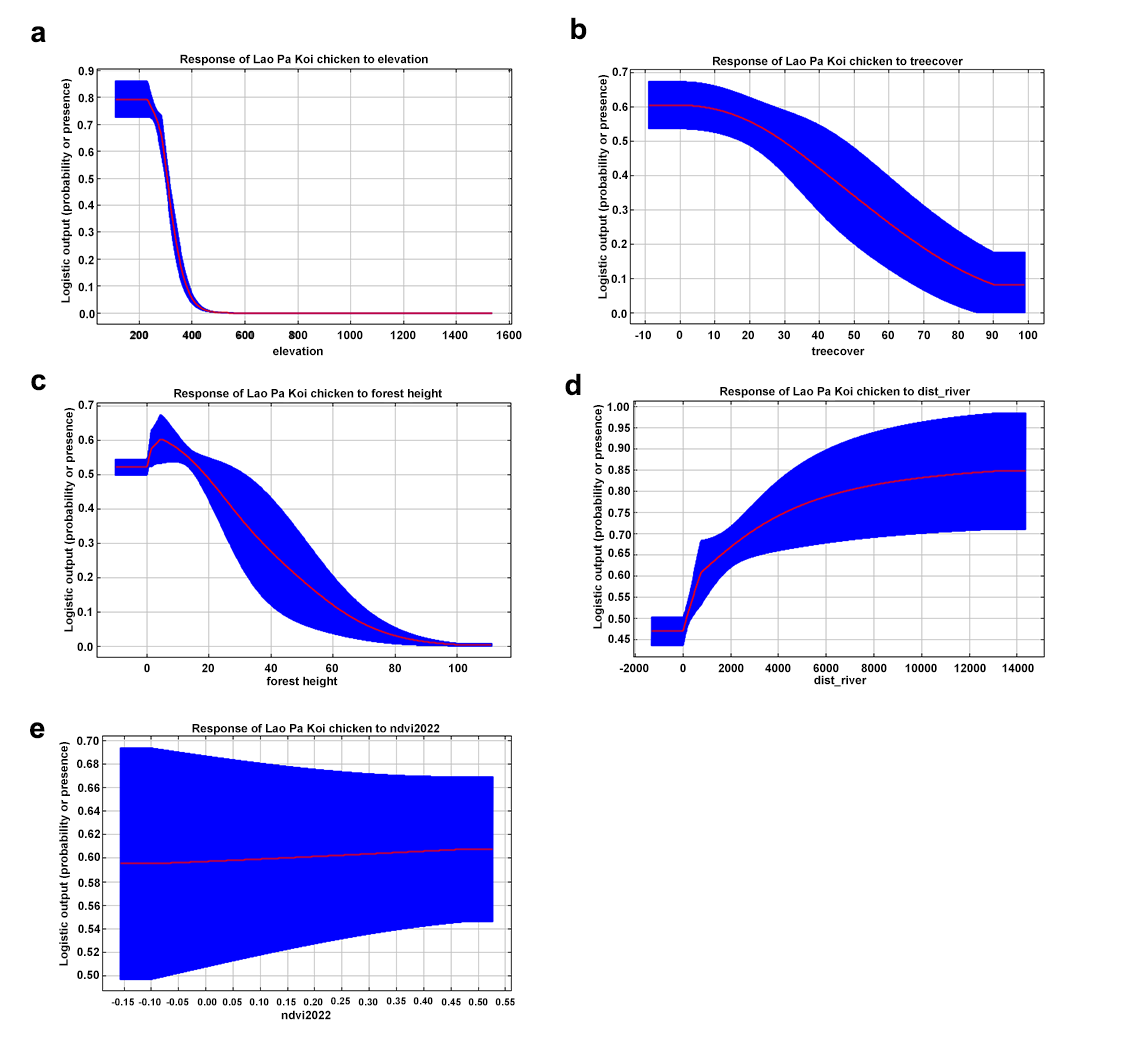

Supplement: S2 Fig — (a) Elevation, (b) Tree canopy cover (b) Forest canopy height, (d) Distance to main river, and (e) Normalized difference vegetation index (NDVI). (TIFF) [file pone.0289983.s002.tiff]

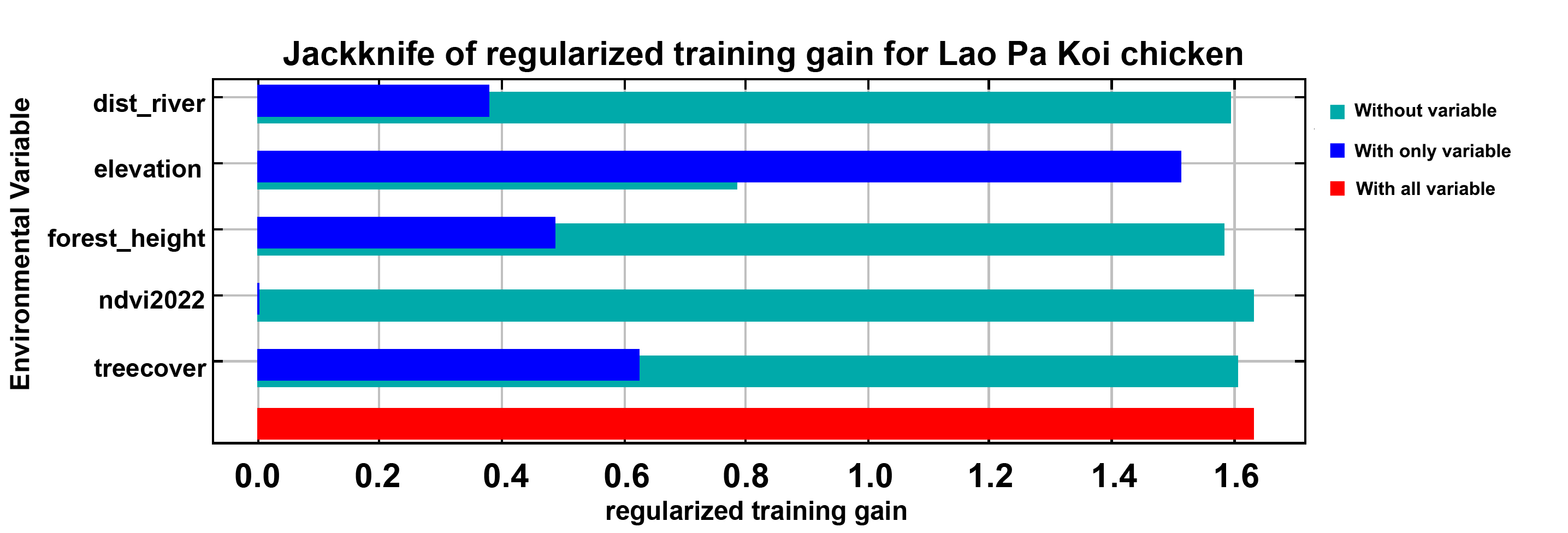

Supplement: S3 Fig — (TIFF) [file pone.0289983.s003.tiff]

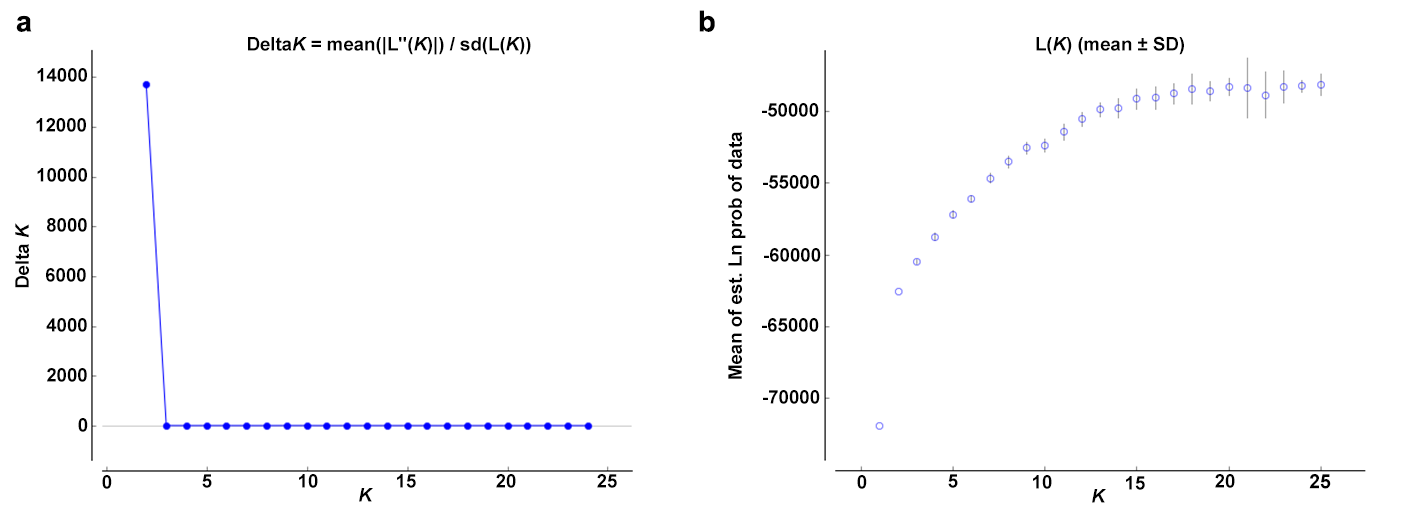

Supplement: S4 Fig — Delta K and L(K) values were calculated using STRUCTURE version 2.3.4 that was parallel run using Structure_threader. (a) Plot of Evanno’s ΔK. (b) Plot of ln P(K). (TIFF) [file pone.0289983.s004.tiff]

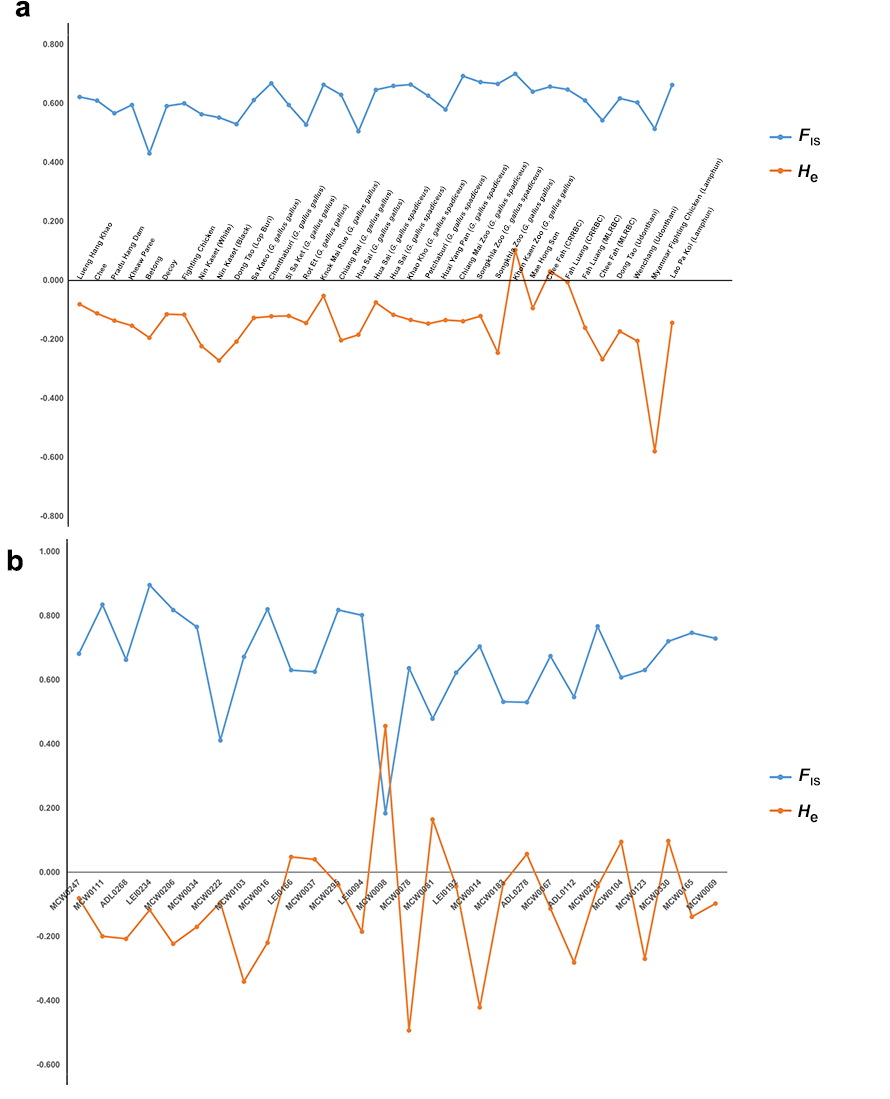

Supplement: S5 Fig — (a) Lao Pa Koi populations. (b) microsatellite loci. (TIFF) [file pone.0289983.s005.tiff]
