# Supplementary material for: Genetic admixture and diversity in Thai domestic chickens revealed through analysis of Lao Pa Koi fighting cocks
Source: PLoS One. 2023 Oct 4;18(10):e0289983. doi: 10.1371/journal.pone.0289983 (PMC10550135; doi:10.1371/journal.pone.0289983)
Supplement: S1 Table — All sequences (accession number LC761600–LC761619) are deposited in the DNA Data Bank of Japan (DDBJ). (DOCX) [file pone.0289983.s006.docx]

**S1 Table.** Specimens collected from 16 smallholder poultry and backyard chicken farms in the Pa Sang district of Lao Pa Koi chickens in Thailand. All sequences (accession number LC761600–LC761619) are deposited in the DNA Data Bank of Japan (DDBJ).

| **No.** | **Abbreviation/Code** | **Locality** | **Sex** | **Mitochondrial D-loop DDBJ accession number** | **Haplogroup** | **Dloop region^*^** |
| --- | --- | --- | --- | --- | --- | --- |
| 1 | LPK01 | 18°26'20.2"N 98°48'52.7"E | Female | LC761600 | CD | 15555– 16289 |
| 2 | LPK02 | 18°26'20.2"N 98°48'52.7"E | Female | LC761601 | B | 15555– 16289 |
| 3 | LPK03 | 18°26'20.2"N 98°48'52.7"E | Female | LC761602 | B | 15555– 16289 |
| 4 | LPK04 | 18°26'20.2"N 98°48'52.7"E | Female | LC761603 | B | 15555– 16289 |
| 5 | LPK05 | 18°26'20.2"N 98°48'52.7"E | Female | LC761604 | B | 15555– 16289 |
| 6 | LPK06 | 18°26'20.2"N 98°48'52.7"E | Female | LC761605 | B | 15555– 16289 |
| 7 | LPK07 | 18°26'20.2"N 98°48'52.7"E | Female | LC761606 | B | 15555– 16289 |
| 8 | LPK08 | 18°26'20.2"N 98°48'52.7"E | Female | LC761607 | CD | 15555– 16289 |
| 9 | LPK09 | 18°26'20.2"N 98°48'52.7"E | Female | LC761608 | B | 15555– 16289 |
| 10 | LPK10 | 18°26'20.2"N 98°48'52.7"E | Female | LC761609 | B | 15555– 16289 |
| 11 | LPK11 | 18°26'20.2"N 98°48'52.7"E | Female | LC761610 | CD | 15555– 16289 |
| 12 | LPK12 | 18°26'20.2"N 98°48'52.7"E | Male | LC761611 | B | 15555– 16289 |
| 13 | LPK13 | 18°26'20.2"N 98°48'52.7"E | Male | LC761612 | B | 15555– 16289 |
| 14 | LPK14 | 18°26'20.2"N 98°48'52.7"E | Male | LC761613 | F | 15555– 16289 |
| 15 | LPK15 | 18°26'20.2"N 98°48'52.7"E | Male | LC761614 | CD | 15555– 16289 |
| 16 | LPK16 | 18°26'20.2"N 98°48'52.7"E | Male | LC761615 | B | 15555– 16289 |
| 17 | LPK17 | 18°26'20.2"N 98°48'52.7"E | Male | LC761616 | B | 15555– 16289 |
| 18 | LPK18 | 18°26'20.2"N 98°48'52.7"E | Male | LC761617 | B | 15555– 16289 |
| 19 | LPK19 | 18°26'20.2"N 98°48'52.7"E | Male | LC761618 | B | 15555– 16289 |
| 20 | LPK20 | 18°26'20.2"N 98°48'52.7"E | Male | LC761619 | B | 15555– 16289 |

^*^Aligned with chicken (*Gallus gallus*) mitochondrion genome (accession NO: NC_053523.1)
