# Supplementary material for: Genetic admixture and diversity in Thai domestic chickens revealed through analysis of Lao Pa Koi fighting cocks
Source: PLoS One. 2023 Oct 4;18(10):e0289983. doi: 10.1371/journal.pone.0289983 (PMC10550135; doi:10.1371/journal.pone.0289983)
Supplement: S2 Table — (DOCX) [file pone.0289983.s007.docx]

**S2 Table.** Microsatellite primers and their nucleotide sequences used for the present study.

| Locus | Chromosome | Annealing temperate (°C) | Dye | Primer sequence 5′ to 3′ | | Size (bp) |
| --- | --- | --- | --- | --- | --- | --- |
|  |  |  |  | Forward | Reverse |  |
| ADL0268 | 1 | 58 | Hex | CTCCACCCCTCTCAGAACTA | CAACTTCCCATCTACCTACT | 106–127 |
| MCW0111 | 1 | 58 | Hex | GCTCCATGTGAAGTGGTTTA | GCTCCATGTGAAGTGGTTTA | 94–120 |
| MCW0247 | 1 | 58 | Fam | GTTGTTCAAAAGAAGATGCATG | TTGCATTAACTGGGCACTTTC | 208–222 |
| LEI0234 | 2 | 58 | Hex | ATGCATCAGATTGGTATTCAA | CGTGGCTGTGAACAAATATG | 218–373 |
| MCW0034 | 2 | 58 | Hex | ATCTTGAAACCTCACAAAGC | TCTTCCAACCTATTTTTAGT | 211–241 |
| MCW0206 | 2 | 58 | Fam | CTTGACAGTGATGCATTAAATG | ACATCTAGAATTGACTGTTCAC | 217–247 |
| LEI0166 | 3 | 58 | Hex | CTCCTGCCCTTAGCTACGCA | TATCCCCTGGCTGGGAGTTT | 338–365 |
| MCW0222 | 3 | 58 | Hex | GCAGTTACATTGAAATGATTCC | TTCTCAAAACACCTAGAAGAC | 218–230 |
| MCW0016 | 3 | 58 | Fam | ATGGCGCAGAAGGCAAAGCGATAT | TGGCTTCTGAAGCAGTTGCTATGG | 126–177 |
| MCW0037 | 3 | 58 | Fam | ACCGGTGCCATCAATTACCTATTA | GAAAGCTCACATGACACTGCGAAA | 149–154 |
| MCW0103 | 3 | 58 | Fam | AACTGCGTTGAGAGTGAATGC | TTTCCTAACTGGATGCTTCTG | 263–270 |
| MCW0295 | 4 | 58 | Fam | ATCACTACAGAACACCCTCTC | TATGTATGCACGCAGATATCC | 82–111 |
| LEI0094 | 4 | 58 | Hex | GATCTCACCAGTATGAGCTGC | TCTCACACTGTAACACAGTGC | 243–281 |
| MCW0078 | 5 | 58 | Hex | CCACACGGAGAGGAGAAGGTCT | TAGCATATGAGTGTACTGAGCTTC | 144–154 |
| MCW0098 | 4 | 58 | Hex | GGCTGCTTTGTGCTCTTCTCG | CGATGGTCGTAATTCTCACGT | 247–257 |
| MCW0081 | 5 | 58 | Fam | GTTGCTGAGAGCCTGGTGCAG | CCTGTATGTGGAATTACTTCTC | 107–146 |
| LEI0192 | 6 | 58 | Hex | TGCCAGAGCTTCAGTCTGT | GTCATTACTGTTATGTTTATTGC | 251–490 |
| MCW0014 | 6 | 58 | Hex | TATTGGCTCTAGGAACTGTC | GAAATGAAGGTAAGACTAGC | 169–202 |
| MCW0183 | 7 | 58 | Hex | ATCCCAGTGTCGAGTATCCGA | TGAGATTTACTGGAGCCTGCC | 293–361 |
| ADL0278 | 8 | 58 | Hex | CCAGCAGTCTACCTTCCTAT | TGTCATCCAAGAACAGTGTG | 118–136 |
| MCW0067 | 10 | 58 | Fam | GCACTACTGTGTGCTGCAGTTT | GAGATGTAGTTGCCACATTCCGAC | 170–182 |
| ADL0112 | 10 | 58 | Hex | GGCTTAAGCTGACCCATTAT | ATCTCAAATGTAATGCGTGC | 132–142 |
| MCW0216 | 13 | 58 | Hex | GGGTTTTACAGGATGGGACG | AGTTTCACTCCCAGGGCTCG | 135–170 |
| MCW0104 | 13 | 58 | Hex | TAGCACAACTCAAGCTGTGAG | AGACTTGCACAGCTGTGACC | 194–249 |
| MCW0123 | 14 | 58 | Hex | CCACTAGAAAAGAACATCCTC | GGCTGATGTAAGAAGGGATGA | 84–106 |
| MCW0330 | 17 | 58 | Hex | TGGACCTCATCAGTCTGACAG | AATGTTCTCATAGAGTTCCTGC | 259–291 |
| MCW0165 | 23 | 58 | Hex | CAGACATGCATGCCCAGATGA | GATCCAGTCCTGCAGGCTGC | 111–115 |
| MCW0069 | 26 | 58 | Hex | GCACTCGAGAAAACTTCCTGCG | ATTGCTTCAGCAAGCATGGGAGGA | 155–178 |

HEX = HEX™ Dye Phosphoramidite, FAM = 6-FAM (6-Carboxyfluorescein)
