# Supplementary material for: Genetic admixture and diversity in Thai domestic chickens revealed through analysis of Lao Pa Koi fighting cocks
Source: PLoS One. 2023 Oct 4;18(10):e0289983. doi: 10.1371/journal.pone.0289983 (PMC10550135; doi:10.1371/journal.pone.0289983)
Supplement: S3 Table — FST, Wright’s F-statistics for subpopulations within the total population. (DOCX) [file pone.0289983.s008.docx]

**S3** **Table.** Genetic differentiation between Lao Pa Koi chickens (the present study) and other domestic chickens reported in our previous studies (Hata et al., 2021, Singchat et al., 2022), based on the mitochondrial D-loop sequence. FST, Wright’s F-statistics for subpopulations within the total population.

|  | **Fah Luang (MLRBC)** | **Chee Fah (MLRBC)** | **Mae Hong Son** | **Chee Fah (CRRBC)** | **Chiang Mai Zoo (*G. gallus spadiceus*)** | **Dong Tao (Udonthani)** | **Fah Luang (CRRBC)** | **Khon Kaen Zoo (*G. gallus gallus*)** | **Lao Pa Koi (Lamphun)** | **Dong Tao (Lop Buri)** | **Betong** | **Nin Kaset (White)** | **Nin Kaset (Black)** | **Lueng Hang Khao** | **Chee** | **Pradu Hang Dam** | **Kheaw Paree** | **Decoy** | **Hua Sai (*G. gallus gallus*)** | **Hua Sai (*G. gallus spadiceus*)** | **Sa Kaeo (*G. gallus gallus*)** | **Chanthaburi (*G. gallus gallus)*** | **Fighting Chicken** | **Si Sa Ket (*G. gallus gallus*)** | **Rot Et (*G. gallus gallus*)** | **Khao Kho (*G. gallus spadiceus*)** | **Chaiyaphum (*G. gallus spadiceus*)** | **Petchaburi (*G. gallus spadiceus*)** | **Huai Yang Pan (*G. gallus spadiceus*)** | **Knok Mai Rue (*G. gallus gallus*)** | **Chiang Rai (*G. gallus gallus*)** | **Myanmar Fighting Chicken (Lamphun)** | **Songkhla Zoo (*G. gallus spadiceus*)** | **Songkhla Zoo (*G. gallus gallus*)** | **Wenchang (Udonthani)** |
| --- | --- | --- | --- | --- | --- | --- | --- | --- | --- | --- | --- | --- | --- | --- | --- | --- | --- | --- | --- | --- | --- | --- | --- | --- | --- | --- | --- | --- | --- | --- | --- | --- | --- | --- | --- |
| Fah Luang (MLRBC) |  |  |  |  |  |  |  |  |  |  |  |  |  |  |  |  |  |  |  |  |  |  |  |  |  |  |  |  |  |  |  |  |  |  |  |
| Chee Fah (MLRBC) | 0.063 |  |  |  |  |  |  |  |  |  |  |  |  |  |  |  |  |  |  |  |  |  |  |  |  |  |  |  |  |  |  |  |  |  |  |
| Mae Hong Son | 0.505 | 0.506 |  |  |  |  |  |  |  |  |  |  |  |  |  |  |  |  |  |  |  |  |  |  |  |  |  |  |  |  |  |  |  |  |  |
| Chee Fah (CRRBC) | 0.063^ns^ | 0.000 | 0.506^ns^ |  |  |  |  |  |  |  |  |  |  |  |  |  |  |  |  |  |  |  |  |  |  |  |  |  |  |  |  |  |  |  |  |
| Chiang Mai Zoo (*G. gallus spadiceus*) | 0.552 | 0.555 | 0.027 | 0.555 |  |  |  |  |  |  |  |  |  |  |  |  |  |  |  |  |  |  |  |  |  |  |  |  |  |  |  |  |  |  |  |
| Dong Tao (Udonthani) | 0.380^ns^ | 0.381^ns^ | 0.043 | 0.3808^ns^ | -0.058 |  |  |  |  |  |  |  |  |  |  |  |  |  |  |  |  |  |  |  |  |  |  |  |  |  |  |  |  |  |  |
| Fah Luang (CRRBC) | 0.034 | 0.226 | 0.499 | 0.226 | 0.532 | 0.368 |  |  |  |  |  |  |  |  |  |  |  |  |  |  |  |  |  |  |  |  |  |  |  |  |  |  |  |  |  |
| Khon Kaen Zoo (*G. gallus gallus*) | 0.212 | 0.213 | 0.197 | 0.213 | 0.031 | 0.042 | 0.202 |  |  |  |  |  |  |  |  |  |  |  |  |  |  |  |  |  |  |  |  |  |  |  |  |  |  |  |  |
| Lao Pa Koi (Lamphun) | 0.482 | 0.483 | 0.004 | 0.483 | -0.062 | -0.030 | 0.471 | 0.115 |  |  |  |  |  |  |  |  |  |  |  |  |  |  |  |  |  |  |  |  |  |  |  |  |  |  |  |
| Dong Tao (Lop Buri) | -0.030 | 0.000 | 0.468 | 0.000 | 0.425 | 0.306 | 0.111 | 0.135 | 0.413 |  |  |  |  |  |  |  |  |  |  |  |  |  |  |  |  |  |  |  |  |  |  |  |  |  |  |
| Betong | 0.206 | 0.211 | 0.280 | 0.211 | 0.128 | 0.129 | 0.196 | 0.025 | 0.215 | 0.148 |  |  |  |  |  |  |  |  |  |  |  |  |  |  |  |  |  |  |  |  |  |  |  |  |  |
| Nin Kaset (White) | 0.998 | 1.000 | 0.327 | 1.000 | 0.435 | 0.387 | 0.997 | 0.549 | 0.338 | 0.999 | 0.545 |  |  |  |  |  |  |  |  |  |  |  |  |  |  |  |  |  |  |  |  |  |  |  |  |
| Nin Kaset (Black) | 0.998 | 0.999 | 0.327 | 0.999 | 0.436 | 0.388 | 0.997 | 0.550 | 0.339 | 0.999 | 0.545 | 0.178 |  |  |  |  |  |  |  |  |  |  |  |  |  |  |  |  |  |  |  |  |  |  |  |
| Lueng Hang Khao | 0.157 | 0.160 | 0.249 | 0.160 | 0.080 | 0.074 | 0.145 | -0.044 | 0.163 | 0.074 | -0.002 | 0.628 | 0.629 |  |  |  |  |  |  |  |  |  |  |  |  |  |  |  |  |  |  |  |  |  |  |
| Chee | 0.013 | 0.021 | 0.386 | 0.021 | 0.292 | 0.215^ns^ | 0.002 | 0.042 | 0.323 | -0.070 | 0.053 | 0.856 | 0.856 | -0.022 |  |  |  |  |  |  |  |  |  |  |  |  |  |  |  |  |  |  |  |  |  |
| Pradu Hang Dam | 0.012 | 0.020 | 0.386 | 0.020 | 0.291 | 0.215 | -0.001 | 0.042 | 0.323 | -0.071 | 0.055 | 0.856 | 0.856 | -0.022 | -0.123 |  |  |  |  |  |  |  |  |  |  |  |  |  |  |  |  |  |  |  |  |
| Kheaw Paree | 0.088 | 0.382 | 0.485 | 0.382 | 0.485 | 0.341 | -0.047 | 0.176 | 0.446 | 0.243^ns^ | 0.170 | 0.997 | 0.997 | 0.115 | -0.033 | -0.033 |  |  |  |  |  |  |  |  |  |  |  |  |  |  |  |  |  |  |  |
| Decoy | 0.314 | 0.321 | 0.149 | 0.321 | -0.050 | -0.031 | 0.289 | -0.099 | 0.046 | 0.171 | -0.047 | 0.655 | 0.655 | -0.110 | 0.008 | 0.010 | 0.231^ns^ |  |  |  |  |  |  |  |  |  |  |  |  |  |  |  |  |  |  |
| Hua Sai (*G. gallus gallus*) | 0.997 | 1.000 | 0.169 | 1.000 | 0.073 | 0.184 | 0.996 | 0.407 | 0.100 | 1.000 | 0.481 | 0.992 | 0.990 | 0.486 | 0.759 | 0.758 | 0.995 | 0.370 |  |  |  |  |  |  |  |  |  |  |  |  |  |  |  |  |  |
| Hua Sai (*G. gallus spadiceus*) | 0.457 | 0.460 | 0.051 | 0.460 | -0.099 | -0.036 | 0.442 | 0.042 | -0.036 | 0.366 | 0.136 | 0.401 | 0.401 | 0.082 | 0.256 | 0.257 | 0.406 | -0.044 | 0.144 |  |  |  |  |  |  |  |  |  |  |  |  |  |  |  |  |
| Sa Kaeo (*G. gallus gallus*) | 0.141 | 0.141 | 0.265 | 0.141 | 0.107 | 0.096 | 0.133 | -0.028 | 0.185 | 0.077 | 0.027 | 0.587 | 0.588 | -0.048 | -0.002 | -0.002 | 0.114 | -0.081 | 0.494 | 0.107 |  |  |  |  |  |  |  |  |  |  |  |  |  |  |  |
| Chanthaburi (*G. gallus gallus)* | 0.470 | 0.472 | 0.062 | 0.472 | -0.063 | 0.013 | 0.462 | 0.112 | -0.007 | 0.417 | 0.211 | 0.324 | 0.325 | 0.167 | 0.325 | 0.325 | 0.442 | 0.055 | 0.132 | -0.033 | 0.184 |  |  |  |  |  |  |  |  |  |  |  |  |  |  |
| Fighting Chicken | 0.122 | 0.124 | 0.282 | 0.124 | 0.130 | 0.112 | 0.114 | -0.022 | 0.205 | 0.055 | 0.008 | 0.619 | 0.619 | -0.054 | -0.026 | -0.026 | 0.090 | -0.083 | 0.523 | 0.126 | -0.033 | 0.206 |  |  |  |  |  |  |  |  |  |  |  |  |  |
| Si Sa Ket (*G. gallus gallus*) | 0.999 | 1.000 | 0.379 | 1.000 | 0.684 | 0.546^ns^ | 0.999 | 0.718 | 0.453 | 1.000 | 0.746 | 0.999 | 0.999 | 0.799 | 0.942 | 0.942 | 0.999 | 0.852 | 1.000 | 0.581 | 0.727 | 0.388 | 0.771 |  |  |  |  |  |  |  |  |  |  |  |  |
| Rot Et (*G. gallus gallus*) | 0.992 | 0.993 | 0.355 | 0.993 | 0.660 | 0.531^ns^ | 0.992 | 0.713 | 0.433 | 0.992 | 0.737 | 0.961 | 0.961 | 0.789 | 0.933^ns^ | 0.933 | 0.991 | 0.835 | 0.938 | 0.560 | 0.721 | 0.384 | 0.764^ns^ | 0.759^ns^ |  |  |  |  |  |  |  |  |  |  |  |
| Khao Kho (*G. gallus spadiceus*) | 0.434 | 0.435 | 0.058 | 0.435 | -0.070 | -0.002 | 0.425 | 0.086 | -0.012 | 0.379 | 0.175 | 0.328 | 0.328 | 0.131 | 0.284 | 0.284 | 0.404 | 0.019 | 0.149 | -0.043 | 0.150 | -0.015 | 0.169 | 0.432 | 0.412 |  |  |  |  |  |  |  |  |  |  |
| Chaiyaphum (*G. gallus spadiceus*) | 0.030 | 0.041 | 0.455 | 0.041 | 0.429 | 0.323 | 0.027 | 0.131 | 0.433 | -0.022 | 0.124 | 0.821 | 0.821 | 0.061 | -0.058 | -0.061 | 0.002 | 0.129 | 0.788 | 0.381 | 0.072 | 0.413 | 0.045 | 0.897 | 0.891 | 0.374 |  |  |  |  |  |  |  |  |  |
| Petchaburi (*G. gallus spadiceus*) | 0.646 | 0.648 | 0.086 | 0.648 | 0.019 | 0.098 | 0.637 | 0.275 | 0.025 | 0.597 | 0.365 | 0.340 | 0.341 | 0.335 | 0.507 | 0.508 | 0.617 | 0.219 | 0.075 | 0.029 | 0.347 | 0.030 | 0.371 | 0.329 | 0.291 | 0.045 | 0.589 |  |  |  |  |  |  |  |  |
| Huai Yang Pan (*G. gallus spadiceus*) | 0.481 | 0.482 | 0.057 | 0.482 | -0.065 | 0.014 | 0.472 | 0.117 | -0.009 | 0.426 | 0.217 | 0.323 | 0.323 | 0.172 | 0.333 | 0.333 | 0.451 | 0.059 | 0.128 | -0.033 | 0.189 | -0.031 | 0.212 | 0.391 | 0.383 | -0.019 | 0.423 | 0.026 |  |  |  |  |  |  |  |
| Knok Mai Rue (*G. gallus gallus*) | 0.684 | 0.685 | 0.102 | 0.685 | 0.061 | 0.143 | 0.677 | 0.325 | 0.061 | 0.643 | 0.415 | 0.342 | 0.342 | 0.392 | 0.559 | 0.559 | 0.661 | 0.293 | 0.105 | 0.085 | 0.396 | 0.045 | 0.423 | 0.255 | 0.216 | 0.071 | 0.623 | -0.009 | 0.039 |  |  |  |  |  |  |
| Chiang Rai (*G. gallus gallus*) | 0.508 | 0.512 | 0.025 | 0.512 | -0.152 | -0.084 | 0.486 | -0.037 | -0.065 | 0.366 | 0.075 | 0.516 | 0.516 | 0.011 | 0.217 | 0.216 | 0.432 | -0.118 | 0.165 | -0.120 | 0.034 | -0.084 | 0.058 | 0.738 | 0.717 | -0.088 | 0.354 | 0.047 | -0.085 | 0.088 |  |  |  |  |  |
| Myanmar Fighting Chicken (Lamphun) | 0.630 | 0.688 | 0.384 | 0.688 | 0.245 | 0.164 | 0.585 | -0.001 | 0.299 | 0.474 | 0.038 | 0.986 | 0.986 | -0.060 | -0.152 | -0.155 | 0.505 | -0.048 | 0.939 | 0.238 | -0.032 | 0.337 | -0.061 | 0.996 | 0.987 | 0.292 | -0.080 | 0.536 | 0.345 | 0.593 | 0.153 |  |  |  |  |
| Songkhla Zoo (*G. gallus spadiceus*) | 0.526 | 0.875 | 0.453 | 0.875 | 0.384 | 0.282 | 0.455 | 0.113 | 0.393 | 0.798 | 0.111 | 0.998 | 0.998 | 0.046 | -0.103 | -0.105 | 0.278 | 0.114 | 0.997 | 0.335 | 0.061 | 0.397 | 0.030 | 1.000 | 0.991 | 0.357 | -0.087 | 0.581 | 0.405 | 0.629 | 0.313 | 0.353 |  |  |  |
| Songkhla Zoo (*G. gallus gallus*) | 0.021 | 0.038 | 0.421 | 0.038^ns^ | 0.371 | 0.270^ns^ | 0.014 | 0.089 | 0.377 | -0.046^ns^ | 0.094 | 0.871 | 0.871 | 0.030 | -0.089 | -0.089 | -0.022 | 0.085 | 0.807 | 0.322 | 0.046 | 0.370 | 0.019 | 0.944 | 0.936^ns^ | 0.332 | -0.051^ns^ | 0.552^ns^ | 0.379^ns^ | 0.596 | 0.298 | -0.127 | -0.116 |  |  |
| Wenchang (Udonthani) | -0.023 | -0.020 | 0.436 | -0.020 | 0.400 | 0.284 | -0.028 | 0.109 | 0.393 | -0.095 | 0.117 | 0.880 | 0.880 | 0.044 | -0.090 | -0.090 | -0.049 | 0.116 | 0.822 | 0.346 | 0.052 | 0.391 | 0.030 | 0.947 | 0.939 | 0.352 | -0.024 | 0.568 | 0.400 | 0.613 | 0.335 | -0.130 | -0.094 | -0.056 |  |

* *p* < 0.05

** *p* < 0.01

ns = not significant
