# Supplementary material for: Genetic admixture and diversity in Thai domestic chickens revealed through analysis of Lao Pa Koi fighting cocks
Source: PLoS One. 2023 Oct 4;18(10):e0289983. doi: 10.1371/journal.pone.0289983 (PMC10550135; doi:10.1371/journal.pone.0289983)
Supplement: S4 Table — Numbers indicate p-values with 110 permutations. (DOCX) [file pone.0289983.s009.docx]

**S4 Table.** Pairwise comparison of linkage disequilibrium of 28 microsatellite lociin Lao Pa Koi chickens . Numbers indicate *p*-values with 110 permutations.

| **Locus** | MCW0111 | MCW0247 | LEI0234 | LEI0166 | MCW0222 | MCW0037 | ADL0268 | MCW0034 | MCW0206 | MCW0016 | MCW0103 | MCW0295 | LEI0094 | MCW0098 | MCW0078 | MCW0081 | LEI0192 | MCW0014 | MCW0183 | MCW0067 | ADL0112 | MCW0216 | MCW0104 | MCW0123 | MCW0330 | MCW0165 | MCW0069 | ADL0278 |
| --- | --- | --- | --- | --- | --- | --- | --- | --- | --- | --- | --- | --- | --- | --- | --- | --- | --- | --- | --- | --- | --- | --- | --- | --- | --- | --- | --- | --- |
| MCW0111 |  |  |  |  |  |  |  |  |  |  |  |  |  |  |  |  |  |  |  |  |  |  |  |  |  |  |  |  |
| MCW0247 | 1.000 |  |  |  |  |  |  |  |  |  |  |  |  |  |  |  |  |  |  |  |  |  |  |  |  |  |  |  |
| LEI0234 | 1.000 | 1.000 |  |  |  |  |  |  |  |  |  |  |  |  |  |  |  |  |  |  |  |  |  |  |  |  |  |  |
| LEI0166 | 0.745 | 0.444 | 1.000 |  |  |  |  |  |  |  |  |  |  |  |  |  |  |  |  |  |  |  |  |  |  |  |  |  |
| MCW0222 | 0.781 | 0.130 | 0.132 | 0.905 |  |  |  |  |  |  |  |  |  |  |  |  |  |  |  |  |  |  |  |  |  |  |  |  |
| MCW0037 | 0.362 | 0.243 | 0.287 | 0.745 | 0.493 |  |  |  |  |  |  |  |  |  |  |  |  |  |  |  |  |  |  |  |  |  |  |  |
| ADL0268 | 0.250 | 0.910 | 0.910 | 0.053 | 0.022 | 0.575 |  |  |  |  |  |  |  |  |  |  |  |  |  |  |  |  |  |  |  |  |  |  |
| MCW0034 | 0.497 | 0.529 | 1.000 | 0.534 | 0.497 | 0.649 | 0.471 |  |  |  |  |  |  |  |  |  |  |  |  |  |  |  |  |  |  |  |  |  |
| MCW0206 | 0.700 | 0.423 | 1.000 | 0.480 | 0.821 | 0.000 | 0.535 | 0.700 |  |  |  |  |  |  |  |  |  |  |  |  |  |  |  |  |  |  |  |  |
| MCW0016 | 0.824 | 0.827 | 1.000 | 0.824 | 0.296 | 1.000 | 0.476 | 0.716 | 0.050 |  |  |  |  |  |  |  |  |  |  |  |  |  |  |  |  |  |  |  |
| MCW0103 | 0.563 | 0.224 | 0.078 | 0.641 | 0.781 | 0.946 | 0.424 | 0.158 | 0.677 | 0.563 |  |  |  |  |  |  |  |  |  |  |  |  |  |  |  |  |  |  |
| MCW0295 | 0.435 | 1.000 | 1.000 | 1.000 | 0.847 | 0.362 | 0.724 | 0.607 | 1.000 | 1.000 | 0.947 |  |  |  |  |  |  |  |  |  |  |  |  |  |  |  |  |  |
| LEI0094 | 0.095 | 0.917 | 1.000 | 0.926 | 0.888 | 0.974 | 0.698 | 0.297 | 0.394 | 0.190 | 0.670 | 0.435 |  |  |  |  |  |  |  |  |  |  |  |  |  |  |  |  |
| MCW0098 | 0.646 | 0.447 | 0.474 | 0.758 | 0.204 | 0.403 | 0.360 | 0.962 | 0.090 | 0.146 | 0.289 | 0.960 | 0.095 |  |  |  |  |  |  |  |  |  |  |  |  |  |  |  |
| MCW0078 | 0.863 | 0.646 | 1.000 | 1.000 | 0.342 | 0.015 | 0.495 | 0.290 | 0.305 | 0.614 | 0.926 | 0.564 | 1.000 | 0.646 |  |  |  |  |  |  |  |  |  |  |  |  |  |  |
| MCW0081 | 0.832 | 0.041 | 1.000 | 0.682 | 1.000 | 0.915 | 0.802 | 0.889 | 0.136 | 0.992 | 0.405 | 0.211 | 0.995 | 0.645 | 0.863 |  |  |  |  |  |  |  |  |  |  |  |  |  |
| LEI0192 | 0.574 | 0.187 | 1.000 | 0.513 | 0.409 | 0.549 | 0.633 | 0.766 | 0.758 | 0.101 | 0.468 | 0.753 | 0.241 | 0.243 | 0.768 | 0.832 |  |  |  |  |  |  |  |  |  |  |  |  |
| MCW0014 | 0.782 | 0.274 | 0.061 | 0.397 | 0.094 | 0.057 | 0.707 | 0.593 | 0.525 | 0.988 | 0.270 | 0.230 | 0.939 | 0.062 | 1.000 | 0.729 | 0.574 |  |  |  |  |  |  |  |  |  |  |  |
| MCW0183 | 0.069 | 0.647 | 0.351 | 0.900 | 0.708 | 0.722 | 0.285 | 0.820 | 0.008 | 0.145 | 0.923 | 0.800 | 0.488 | 0.060 | 0.553 | 0.681 | 0.562 | 0.782 |  |  |  |  |  |  |  |  |  |  |
| MCW0067 | 0.751 | 0.964 | 1.000 | 0.987 | 0.499 | 0.199 | 0.828 | 0.480 | 0.205 | 0.929 | 0.892 | 0.157 | 0.614 | 0.154 | 0.754 | 0.287 | 0.821 | 0.131 | 0.610 |  |  |  |  |  |  |  |  |  |
| ADL0112 | 0.967 | 0.509 | 0.581 | 0.048 | 0.543 | 0.736 | 0.379 | 0.585 | 0.734 | 0.008 | 0.797 | 1.000 | 0.285 | 0.184 | 0.178 | 0.906 | 0.180 | 0.074 | 0.202 | 0.751 |  |  |  |  |  |  |  |  |
| MCW0216 | 0.702 | 0.877 | 1.000 | 0.603 | 0.402 | 0.421 | 0.637 | 0.544 | 0.100 | 0.524 | 0.165 | 0.439 | 0.239 | 0.807 | 0.103 | 0.576 | 0.284 | 0.268 | 0.859 | 0.844 | 0.967 |  |  |  |  |  |  |  |
| MCW0104 | 0.282 | 0.022 | 1.000 | 0.140 | 0.558 | 0.968 | 0.865 | 0.259 | 0.069 | 0.544 | 0.057 | 0.739 | 0.793 | 0.476 | 0.821 | 0.093 | 0.391 | 0.251 | 0.374 | 0.157 | 0.582 | 0.702 |  |  |  |  |  |  |
| MCW0123 | 0.258 | 0.760 | 0.066 | 0.736 | 0.424 | 0.811 | 0.051 | 0.931 | 0.596 | 0.473 | 0.043 | 0.854 | 0.374 | 0.187 | 0.930 | 0.275 | 0.186 | 0.028 | 0.690 | 0.363 | 0.191 | 0.364 | 0.282 |  |  |  |  |  |
| MCW0330 | 0.883 | 0.744 | 1.000 | 0.245 | 0.141 | 0.873 | 0.270 | 0.766 | 0.212 | 1.000 | 0.149 | 1.000 | 1.000 | 0.893 | 0.664 | 0.447 | 0.937 | 0.794 | 0.427 | 0.185 | 0.914 | 0.792 | 0.567 | 0.258 |  |  |  |  |
| MCW0165 | 0.608 | 0.639 | 1.000 | 0.039 | 0.557 | 0.668 | 0.323 | 0.654 | 0.888 | 0.860 | 0.645 | 1.000 | 0.352 | 0.318 | 0.234 | 0.877 | 0.175 | 0.408 | 0.552 | 0.340 | 0.018 | 1.000 | 0.614 | 0.384 | 0.883 |  |  |  |
| MCW0069 | 0.658 | 0.768 | 1.000 | 0.361 | 0.271 | 0.942 | 0.985 | 0.549 | 0.906 | 0.242 | 0.123 | 0.583 | 1.000 | 0.935 | 0.821 | 0.175 | 0.978 | 0.766 | 0.791 | 0.770 | 0.431 | 1.000 | 0.267 | 0.896 | 0.538 | 0.608 |  |  |
| ADL0278 | 0.722 | 0.458 | 0.530 | 0.890 | 1.000 | 0.965 | 0.309 | 0.394 | 0.254 | 0.120 | 0.002 | 0.125 | 0.016 | 0.508 | 0.794 | 0.170 | 0.750 | 0.671 | 0.069 | 0.722 | 1.000 | 0.068 | 0.281 | 0.008 | 0.669 | 0.973 | 0.954 |  |
