# Supplementary material for: Genetic admixture and diversity in Thai domestic chickens revealed through analysis of Lao Pa Koi fighting cocks
Source: PLoS One. 2023 Oct 4;18(10):e0289983. doi: 10.1371/journal.pone.0289983 (PMC10550135; doi:10.1371/journal.pone.0289983)
Supplement: S5 Table — (DOCX) [file pone.0289983.s010.docx]

**S5 Table.** Genetic diversity of Lao Pa Koi chickens based on 28 microsatellite loci.

| **Populations** | **Locus** | ***N*_a_** | ***AR*** | ***N*_e_*_a_*** | ***I*** | ***H*_o_** | ***H*_e_** | ***PIC*** | ***F*** |
| --- | --- | --- | --- | --- | --- | --- | --- | --- | --- |
| **Lamphun** | MCW0247 | 4.000 | 4.000 | 3.139 | 1.252 | 0.737 | 0.681 | 0.628 | -0.081 |
|  | MCW0111 | 9.000 | 8.899 | 6.015 | 1.952 | 1.000 | 0.834 | 0.814 | -0.199 |
|  | ADL0268 | 7.000 | 6.897 | 2.963 | 1.404 | 0.800 | 0.663 | 0.626 | -0.208 |
|  | LEI0234 | 14.000 | 13.747 | 9.524 | 2.422 | 1.000 | 0.895 | 0.886 | -0.117 |
|  | MCW0206 | 8.000 | 7.900 | 5.479 | 1.837 | 1.000 | 0.818 | 0.793 | -0.223 |
|  | MCW0034 | 10.000 | 10.000 | 4.247 | 1.815 | 0.895 | 0.765 | 0.742 | -0.170 |
|  | MCW0222 | 3.000 | 2.950 | 1.699 | 0.672 | 0.450 | 0.411 | 0.345 | -0.094 |
|  | MCW0103 | 4.000 | 4.000 | 3.042 | 1.213 | 0.900 | 0.671 | 0.609 | -0.341 |
|  | MCW0016 | 9.000 | 8.897 | 5.556 | 1.906 | 1.000 | 0.820 | 0.798 | -0.220 |
|  | LEI0166 | 7.000 | 6.899 | 2.703 | 1.373 | 0.600 | 0.630 | 0.603 | 0.048 |
|  | MCW0037 | 3.000 | 3.000 | 2.667 | 1.040 | 0.600 | 0.625 | 0.555 | 0.040 |
|  | MCW0295 | 8.000 | 7.899 | 5.479 | 1.832 | 0.850 | 0.818 | 0.793 | -0.040 |
|  | LEI0094 | 10.000 | 9.799 | 5.031 | 1.885 | 0.950 | 0.801 | 0.777 | -0.186 |
|  | MCW0098 | 3.000 | 2.950 | 1.225 | 0.381 | 0.100 | 0.184 | 0.174 | 0.456 |
|  | MCW0078 | 3.000 | 3.000 | 2.749 | 1.055 | 0.950 | 0.636 | 0.564 | -0.493 |
|  | MCW0081 | 5.000 | 4.949 | 1.918 | 0.971 | 0.400 | 0.479 | 0.448 | 0.164 |
|  | LEI0192 | 12.000 | 11.646 | 2.649 | 1.596 | 0.650 | 0.623 | 0.610 | -0.044 |
|  | MCW0014 | 7.000 | 6.900 | 3.376 | 1.482 | 1.000 | 0.704 | 0.663 | -0.421 |
|  | MCW0183 | 6.000 | 5.897 | 2.133 | 1.086 | 0.550 | 0.531 | 0.492 | -0.035 |
|  | ADL0278 | 5.000 | 4.899 | 2.128 | 0.983 | 0.500 | 0.530 | 0.467 | 0.057 |
|  | MCW0067 | 4.000 | 4.000 | 3.065 | 1.241 | 0.750 | 0.674 | 0.622 | -0.113 |
|  | ADL0112 | 4.000 | 3.900 | 2.204 | 0.890 | 0.700 | 0.546 | 0.444 | -0.281 |
|  | MCW0216 | 7.000 | 6.900 | 4.278 | 1.615 | 0.800 | 0.766 | 0.730 | -0.044 |
|  | MCW0104 | 5.000 | 4.950 | 2.548 | 1.187 | 0.550 | 0.608 | 0.565 | 0.095 |
|  | MCW0123 | 5.000 | 4.996 | 2.703 | 1.175 | 0.800 | 0.630 | 0.560 | -0.270 |
|  | MCW0330 | 5.000 | 4.997 | 3.571 | 1.375 | 0.650 | 0.720 | 0.669 | 0.097 |
|  | MCW0165 | 7.000 | 6.850 | 3.941 | 1.542 | 0.850 | 0.746 | 0.705 | -0.139 |
|  | MCW0069 | 8.000 | 7.799 | 3.687 | 1.545 | 0.800 | 0.729 | 0.687 | -0.098 |
|  | Mean | 6.500 | 6.411 | 3.561 | 1.383 | 0.744 | 0.662 | 0.620 | -0.102 |
|  | SE | 0.536 | 0.524 | 0.325 | 0.083 | 0.042 | 0.028 | 0.029 | 0.036 |
