# Supplementary material for: Genetic admixture and diversity in Thai domestic chickens revealed through analysis of Lao Pa Koi fighting cocks
Source: PLoS One. 2023 Oct 4;18(10):e0289983. doi: 10.1371/journal.pone.0289983 (PMC10550135; doi:10.1371/journal.pone.0289983)
Supplement: S6 Table — (DOCX) [file pone.0289983.s011.docx]

**S6 Table.** Pairwise genetic relatedness (*r*) among Lao Pa Koi chickens.

| **Sample 1** | **Sample 2** | ***r*** |
| --- | --- | --- |
| KOI01_LP | KOI02_LP | -0.067 |
| KOI01_LP | KOI03_LP | -0.019 |
| KOI02_LP | KOI03_LP | -0.057 |
| KOI01_LP | KOI04_LP | 0.019 |
| KOI02_LP | KOI04_LP | -0.008 |
| KOI03_LP | KOI04_LP | 0.009 |
| KOI01_LP | KOI05_LP | -0.054 |
| KOI02_LP | KOI05_LP | 0.105 |
| KOI03_LP | KOI05_LP | -0.021 |
| KOI04_LP | KOI05_LP | 0.001 |
| KOI01_LP | KOI06_LP | -0.012 |
| KOI02_LP | KOI06_LP | -0.028 |
| KOI03_LP | KOI06_LP | 0.020 |
| KOI04_LP | KOI06_LP | 0.006 |
| KOI05_LP | KOI06_LP | -0.002 |
| KOI01_LP | KOI07_LP | -0.023 |
| KOI02_LP | KOI07_LP | -0.034 |
| KOI03_LP | KOI07_LP | -0.005 |
| KOI04_LP | KOI07_LP | -0.078 |
| KOI05_LP | KOI07_LP | -0.022 |
| KOI06_LP | KOI07_LP | 0.062 |
| KOI01_LP | KOI08_LP | 0.042 |
| KOI02_LP | KOI08_LP | -0.057 |
| KOI03_LP | KOI08_LP | -0.040 |
| KOI04_LP | KOI08_LP | 0.022 |
| KOI05_LP | KOI08_LP | -0.038 |
| KOI06_LP | KOI08_LP | -0.038 |
| KOI07_LP | KOI08_LP | -0.036 |
| KOI01_LP | KOI09_LP | -0.035 |
| KOI02_LP | KOI09_LP | -0.014 |
| KOI03_LP | KOI09_LP | 0.003 |
| KOI04_LP | KOI09_LP | -0.084 |
| KOI05_LP | KOI09_LP | -0.008 |
| KOI06_LP | KOI09_LP | -0.002 |
| KOI07_LP | KOI09_LP | 0.066 |
| KOI08_LP | KOI09_LP | -0.063 |
| KOI01_LP | KOI10_LP | -0.019 |
| KOI02_LP | KOI10_LP | -0.064 |
| KOI03_LP | KOI10_LP | -0.073 |
| KOI04_LP | KOI10_LP | -0.077 |
| KOI05_LP | KOI10_LP | -0.074 |
| KOI06_LP | KOI10_LP | -0.016 |
| KOI07_LP | KOI10_LP | 0.040 |
| KOI08_LP | KOI10_LP | -0.043 |
| KOI09_LP | KOI10_LP | -0.052 |
| KOI01_LP | KOI11_LP | -0.013 |
| KOI02_LP | KOI11_LP | -0.047 |
| KOI03_LP | KOI11_LP | -0.017 |
| KOI04_LP | KOI11_LP | -0.092 |
| KOI05_LP | KOI11_LP | -0.008 |
| KOI06_LP | KOI11_LP | 0.012 |
| KOI07_LP | KOI11_LP | -0.037 |
| KOI08_LP | KOI11_LP | -0.066 |
| KOI09_LP | KOI11_LP | -0.028 |
| KOI10_LP | KOI11_LP | -0.070 |
| KOI01_LP | KOI12_LP | -0.002 |
| KOI02_LP | KOI12_LP | -0.038 |
| KOI03_LP | KOI12_LP | -0.014 |
| KOI04_LP | KOI12_LP | -0.018 |
| KOI05_LP | KOI12_LP | -0.030 |
| KOI06_LP | KOI12_LP | -0.005 |
| KOI07_LP | KOI12_LP | 0.002 |
| KOI08_LP | KOI12_LP | -0.023 |
| KOI09_LP | KOI12_LP | -0.026 |
| KOI10_LP | KOI12_LP | 0.002 |
| KOI11_LP | KOI12_LP | -0.012 |
| KOI01_LP | KOI13_LP | 0.051 |
| KOI02_LP | KOI13_LP | -0.037 |
| KOI03_LP | KOI13_LP | 0.010 |
| KOI04_LP | KOI13_LP | 0.024 |
| KOI05_LP | KOI13_LP | -0.008 |
| KOI06_LP | KOI13_LP | -0.041 |
| KOI07_LP | KOI13_LP | -0.026 |
| KOI08_LP | KOI13_LP | -0.028 |
| KOI09_LP | KOI13_LP | -0.046 |
| KOI10_LP | KOI13_LP | -0.040 |
| KOI11_LP | KOI13_LP | -0.052 |
| KOI12_LP | KOI13_LP | 0.011 |
| KOI01_LP | KOI14_LP | 0.006 |
| KOI02_LP | KOI14_LP | -0.019 |
| KOI03_LP | KOI14_LP | 0.002 |
| KOI04_LP | KOI14_LP | 0.005 |
| KOI05_LP | KOI14_LP | -0.035 |
| KOI06_LP | KOI14_LP | -0.022 |
| KOI07_LP | KOI14_LP | -0.008 |
| KOI08_LP | KOI14_LP | -0.017 |
| KOI09_LP | KOI14_LP | 0.005 |
| KOI10_LP | KOI14_LP | -0.019 |
| KOI11_LP | KOI14_LP | -0.064 |
| KOI12_LP | KOI14_LP | 0.021 |
| KOI13_LP | KOI14_LP | -0.029 |
| KOI01_LP | KOI15_LP | -0.048 |
| KOI02_LP | KOI15_LP | -0.038 |
| KOI03_LP | KOI15_LP | -0.048 |
| KOI04_LP | KOI15_LP | -0.039 |
| KOI05_LP | KOI15_LP | -0.034 |
| KOI06_LP | KOI15_LP | -0.072 |
| KOI07_LP | KOI15_LP | -0.025 |
| KOI08_LP | KOI15_LP | -0.028 |
| KOI09_LP | KOI15_LP | -0.036 |
| KOI10_LP | KOI15_LP | -0.024 |
| KOI11_LP | KOI15_LP | -0.047 |
| KOI12_LP | KOI15_LP | -0.052 |
| KOI13_LP | KOI15_LP | -0.042 |
| KOI14_LP | KOI15_LP | -0.010 |
| KOI01_LP | KOI16_LP | -0.028 |
| KOI02_LP | KOI16_LP | 0.066 |
| KOI03_LP | KOI16_LP | -0.076 |
| KOI04_LP | KOI16_LP | -0.074 |
| KOI05_LP | KOI16_LP | 0.055 |
| KOI06_LP | KOI16_LP | -0.051 |
| KOI07_LP | KOI16_LP | -0.014 |
| KOI08_LP | KOI16_LP | -0.063 |
| KOI09_LP | KOI16_LP | 0.054 |
| KOI10_LP | KOI16_LP | -0.011 |
| KOI11_LP | KOI16_LP | -0.018 |
| KOI12_LP | KOI16_LP | 0.023 |
| KOI13_LP | KOI16_LP | -0.028 |
| KOI14_LP | KOI16_LP | -0.035 |
| KOI15_LP | KOI16_LP | -0.057 |
| KOI01_LP | KOI17_LP | -0.056 |
| KOI02_LP | KOI17_LP | -0.026 |
| KOI03_LP | KOI17_LP | -0.002 |
| KOI04_LP | KOI17_LP | 0.040 |
| KOI05_LP | KOI17_LP | -0.058 |
| KOI06_LP | KOI17_LP | -0.037 |
| KOI07_LP | KOI17_LP | -0.031 |
| KOI08_LP | KOI17_LP | -0.075 |
| KOI09_LP | KOI17_LP | -0.044 |
| KOI10_LP | KOI17_LP | -0.053 |
| KOI11_LP | KOI17_LP | -0.007 |
| KOI12_LP | KOI17_LP | -0.037 |
| KOI13_LP | KOI17_LP | -0.049 |
| KOI14_LP | KOI17_LP | -0.020 |
| KOI15_LP | KOI17_LP | 0.005 |
| KOI16_LP | KOI17_LP | -0.042 |
| KOI01_LP | KOI18_LP | -0.087 |
| KOI02_LP | KOI18_LP | -0.027 |
| KOI03_LP | KOI18_LP | -0.032 |
| KOI04_LP | KOI18_LP | -0.023 |
| KOI05_LP | KOI18_LP | -0.024 |
| KOI06_LP | KOI18_LP | -0.038 |
| KOI07_LP | KOI18_LP | -0.033 |
| KOI08_LP | KOI18_LP | -0.030 |
| KOI09_LP | KOI18_LP | -0.039 |
| KOI10_LP | KOI18_LP | -0.008 |
| KOI11_LP | KOI18_LP | -0.017 |
| KOI12_LP | KOI18_LP | -0.005 |
| KOI13_LP | KOI18_LP | -0.022 |
| KOI14_LP | KOI18_LP | -0.050 |
| KOI15_LP | KOI18_LP | -0.017 |
| KOI16_LP | KOI18_LP | -0.048 |
| KOI17_LP | KOI18_LP | 0.009 |
| KOI01_LP | KOI19_LP | -0.018 |
| KOI02_LP | KOI19_LP | -0.013 |
| KOI03_LP | KOI19_LP | -0.032 |
| KOI04_LP | KOI19_LP | -0.052 |
| KOI05_LP | KOI19_LP | -0.032 |
| KOI06_LP | KOI19_LP | -0.062 |
| KOI07_LP | KOI19_LP | -0.005 |
| KOI08_LP | KOI19_LP | 0.023 |
| KOI09_LP | KOI19_LP | -0.037 |
| KOI10_LP | KOI19_LP | -0.074 |
| KOI11_LP | KOI19_LP | -0.075 |
| KOI12_LP | KOI19_LP | -0.015 |
| KOI13_LP | KOI19_LP | -0.038 |
| KOI14_LP | KOI19_LP | 0.015 |
| KOI15_LP | KOI19_LP | -0.080 |
| KOI16_LP | KOI19_LP | -0.025 |
| KOI17_LP | KOI19_LP | -0.054 |
| KOI18_LP | KOI19_LP | -0.031 |
| KOI01_LP | KOI20_LP | -0.032 |
| KOI02_LP | KOI20_LP | -0.027 |
| KOI03_LP | KOI20_LP | -0.004 |
| KOI04_LP | KOI20_LP | -0.057 |
| KOI05_LP | KOI20_LP | -0.074 |
| KOI06_LP | KOI20_LP | -0.031 |
| KOI07_LP | KOI20_LP | -0.046 |
| KOI08_LP | KOI20_LP | -0.062 |
| KOI09_LP | KOI20_LP | 0.002 |
| KOI10_LP | KOI20_LP | -0.055 |
| KOI11_LP | KOI20_LP | 0.008 |
| KOI12_LP | KOI20_LP | 0.012 |
| KOI13_LP | KOI20_LP | -0.067 |
| KOI14_LP | KOI20_LP | -0.017 |
| KOI15_LP | KOI20_LP | -0.042 |
| KOI16_LP | KOI20_LP | -0.025 |
| KOI17_LP | KOI20_LP | -0.019 |
| KOI18_LP | KOI20_LP | -0.038 |
| KOI19_LP | KOI20_LP | -0.008 |
