# Supplementary material for: Genetic admixture and diversity in Thai domestic chickens revealed through analysis of Lao Pa Koi fighting cocks
Source: PLoS One. 2023 Oct 4;18(10):e0289983. doi: 10.1371/journal.pone.0289983 (PMC10550135; doi:10.1371/journal.pone.0289983)
Supplement: S7 Table — (DOCX) [file pone.0289983.s012.docx]

**S7 Table.** Inbreeding coefficients (*F*_IS_) of each individual of Lao Pa Koi chickens.

| **Individual** | ***F*_IS_** |
| --- | --- |
| KOI01_LP | -0.1836 |
| KOI02_LP | -0.1149 |
| KOI03_LP | -0.1243 |
| KOI04_LP | -0.132 |
| KOI05_LP | -0.1185 |
| KOI06_LP | -0.1119 |
| KOI07_LP | -0.1707 |
| KOI08_LP | -0.1415 |
| KOI09_LP | -0.1534 |
| KOI10_LP | -0.1741 |
| KOI11_LP | -0.1783 |
| KOI12_LP | -0.1471 |
| KOI13_LP | -0.129 |
| KOI14_LP | -0.135 |
| KOI15_LP | -0.1183 |
| KOI16_LP | -0.1274 |
| KOI17_LP | -0.1501 |
| KOI18_LP | -0.1544 |
| KOI19_LP | -0.1513 |
| KOI20_LP | -0.1763 |
