# Supplementary material for: Genetic admixture and diversity in Thai domestic chickens revealed through analysis of Lao Pa Koi fighting cocks
Source: PLoS One. 2023 Oct 4;18(10):e0289983. doi: 10.1371/journal.pone.0289983 (PMC10550135; doi:10.1371/journal.pone.0289983)
Supplement: S8 Table — FST, Wright’s F-statistics for subpopulations within the total population. (DOCX) [file pone.0289983.s013.docx]

**S8** **Table.** Genetic differentiation between Lao Pa Koi chickens (the present study) and other domestic chickens reported in our previous studies (Hata et al., 2021, Singchat et al., 2022, Budi et al., 2023, and Wongloet et al., 2023), based on 28 microsatellite loci. FST, Wright’s F-statistics for subpopulations within the total population.

|  | **Lueng Hang Khao** | **Chee** | **Pradu Hang Dam** | **Kheaw Paree** | **Betong** | **Decoy** | **Fighting Chicken** | **Nin Kaset (White)** | **Nin Kaset (Black)** | **Dong Tao (Lop Buri)** | **Sa Kaeo (*G. gallus gallus*)** | **Chanthaburi (*G. gallus gallus)*** | **Si Sa Ket (*G. gallus gallus*)** | **Rot Et (*G. gallus gallus*)** | **Knok Mai Rue (*G. gallus gallus*)** | **Chiang Rai (*G. gallus gallus*)** | **Hua Sai (*G. gallus gallus*)** | **Hua Sai (*G. gallus spadiceus*)** | **Khao Kho (*G. gallus spadiceus*)** | **Chaiyaphum (*G. gallus spadiceus*)** | **Petchaburi (*G. gallus spadiceus*)** | **Huai Yang Pan (*G. gallus spadiceus*)** | **Chiang Mai Zoo (*G. gallus spadiceus*)** | **Songkhla Zoo (*G. gallus gallus*)** | **Songkhla Zoo (*G. gallus spadiceus*)** | **Khon Kaen Zoo (*G. gallus gallus*)** | **Mae Hong Son** | **Chee Fah (CRRBC)** | **Fah Luang (CRRBC)** | **Fah Luang (MLRBC)** | **Chee Fah (MLRBC)** | **Dong Tao (Udonthani)** | **Wenchang (Udonthani)** | **Myanmar Fighting Chicken (Lamphun)** | **Lao Pa Koi (Lamphun)** |
| --- | --- | --- | --- | --- | --- | --- | --- | --- | --- | --- | --- | --- | --- | --- | --- | --- | --- | --- | --- | --- | --- | --- | --- | --- | --- | --- | --- | --- | --- | --- | --- | --- | --- | --- | --- |
| Lueng Hang Khao | 0.000 |  |  |  |  |  |  |  |  |  |  |  |  |  |  |  |  |  |  |  |  |  |  |  |  |  |  |  |  |  |  |  |  |  |  |
| Chee | 0.042^*^ | 0.000 |  |  |  |  |  |  |  |  |  |  |  |  |  |  |  |  |  |  |  |  |  |  |  |  |  |  |  |  |  |  |  |  |  |
| Pradu Hang Dam | 0.035^**^ | 0.094^**^ | 0.000 |  |  |  |  |  |  |  |  |  |  |  |  |  |  |  |  |  |  |  |  |  |  |  |  |  |  |  |  |  |  |  |  |
| Kheaw Paree | 0.008^ns^ | 0.050^*^ | 0.016^ns^ | 0.000 |  |  |  |  |  |  |  |  |  |  |  |  |  |  |  |  |  |  |  |  |  |  |  |  |  |  |  |  |  |  |  |
| Betong | 0.298^**^ | 0.337^**^ | 0.350^**^ | 0.325^**^ | 0.000 |  |  |  |  |  |  |  |  |  |  |  |  |  |  |  |  |  |  |  |  |  |  |  |  |  |  |  |  |  |  |
| Decoy | 0.005^ns^ | 0.029^ns^ | 0.051^*^ | -0.010^ns^ | 0.280^**^ | 0.000 |  |  |  |  |  |  |  |  |  |  |  |  |  |  |  |  |  |  |  |  |  |  |  |  |  |  |  |  |  |
| Fighting Chicken | 0.088^**^ | 0.112^**^ | 0.097^**^ | 0.105^**^ | 0.305^**^ | 0.065^**^ | 0.000 |  |  |  |  |  |  |  |  |  |  |  |  |  |  |  |  |  |  |  |  |  |  |  |  |  |  |  |  |
| Nin Kaset (White) | 0.187^**^ | 0.209^**^ | 0.239^**^ | 0.210^**^ | 0.249^**^ | 0.128^**^ | 0.236 | 0.000 |  |  |  |  |  |  |  |  |  |  |  |  |  |  |  |  |  |  |  |  |  |  |  |  |  |  |  |
| Nin Kaset (Black) | 0.243^**^ | 0.246^**^ | 0.271^**^ | 0.272^**^ | 0.323^**^ | 0.206^**^ | 0.240 | 0.173 | 0.000 |  |  |  |  |  |  |  |  |  |  |  |  |  |  |  |  |  |  |  |  |  |  |  |  |  |  |
| Dong Tao (Lop Buri) | 0.160^**^ | 0.178^**^ | 0.223^**^ | 0.193^**^ | 0.195^**^ | 0.146^**^ | 0.203 | 0.136 | 0.194 | 0.000 |  |  |  |  |  |  |  |  |  |  |  |  |  |  |  |  |  |  |  |  |  |  |  |  |  |
| Sa Kaeo (*G. gallus gallus*) | 0.094^**^ | 0.136^**^ | 0.146^**^ | 0.099^**^ | 0.320^**^ | 0.064^**^ | 0.197 | 0.219 | 0.282 | 0.206 | 0.000 |  |  |  |  |  |  |  |  |  |  |  |  |  |  |  |  |  |  |  |  |  |  |  |  |
| Chanthaburi (*G. gallus gallus)* | 0.138^**^ | 0.154^**^ | 0.198^**^ | 0.164^**^ | 0.334^**^ | 0.089^**^ | 0.214 | 0.198 | 0.230 | 0.195 | 0.153 | 0.000 |  |  |  |  |  |  |  |  |  |  |  |  |  |  |  |  |  |  |  |  |  |  |  |
| Si Sa Ket (*G. gallus gallus*) | 0.168^**^ | 0.156^**^ | 0.172^**^ | 0.162^**^ | 0.363^**^ | 0.106^**^ | 0.144 | 0.217 | 0.245 | 0.243 | 0.199 | 0.185 | 0.000 |  |  |  |  |  |  |  |  |  |  |  |  |  |  |  |  |  |  |  |  |  |  |
| Rot Et (*G. gallus gallus*) | 0.184^**^ | 0.189^**^ | 0.198^**^ | 0.212^**^ | 0.416^**^ | 0.167^**^ | 0.139 | 0.275 | 0.223 | 0.293 | 0.223 | 0.213 | 0.120 | 0.000 |  |  |  |  |  |  |  |  |  |  |  |  |  |  |  |  |  |  |  |  |  |
| Knok Mai Rue (*G. gallus gallus*) | 0.080^**^ | 0.077^**^ | 0.105^**^ | 0.106^**^ | 0.283^**^ | 0.029^ns^ | 0.059 | 0.171 | 0.190 | 0.150 | 0.150 | 0.134 | 0.104 | 0.118 | 0.000 |  |  |  |  |  |  |  |  |  |  |  |  |  |  |  |  |  |  |  |  |
| Chiang Rai (*G. gallus gallus*) | 0.159^**^ | 0.165^**^ | 0.187^**^ | 0.186^**^ | 0.313^**^ | 0.119^**^ | 0.141 | 0.225 | 0.217 | 0.168 | 0.198 | 0.164 | 0.200 | 0.221 | 0.107 | 0.000 |  |  |  |  |  |  |  |  |  |  |  |  |  |  |  |  |  |  |  |
| Hua Sai (*G. gallus gallus*) | 0.100^**^ | 0.148^**^ | 0.173^**^ | 0.141^**^ | 0.229^**^ | 0.096^*^ | 0.134 | 0.177 | 0.254 | 0.070^ns^ | 0.138 | 0.140 | 0.154 | 0.252 | 0.091 | 0.176 | 0.000 |  |  |  |  |  |  |  |  |  |  |  |  |  |  |  |  |  |  |
| Hua Sai (*G. gallus spadiceus*) | 0.086^**^ | 0.076^**^ | 0.119^**^ | 0.100^**^ | 0.249^**^ | 0.068^**^ | 0.105 | 0.152 | 0.223 | 0.126 | 0.154 | 0.173 | 0.191 | 0.213 | 0.080 | 0.122 | 0.062^ns^ | 0.000 |  |  |  |  |  |  |  |  |  |  |  |  |  |  |  |  |  |
| Khao Kho (*G. gallus spadiceus*) | 0.144^**^ | 0.117^**^ | 0.168^**^ | 0.162^**^ | 0.313^**^ | 0.111^**^ | 0.153 | 0.195 | 0.214 | 0.193 | 0.168 | 0.165 | 0.166 | 0.172 | 0.054 | 0.144 | 0.158 | 0.116 | 0.000 |  |  |  |  |  |  |  |  |  |  |  |  |  |  |  |  |
| Chaiyaphum (*G. gallus spadiceus*) | 0.152^**^ | 0.165^**^ | 0.185^**^ | 0.162^**^ | 0.308^**^ | 0.071^**^ | 0.157 | 0.199 | 0.236 | 0.184 | 0.183 | 0.138 | 0.121 | 0.167 | 0.099 | 0.144 | 0.132 | 0.154 | 0.132 | 0.000 |  |  |  |  |  |  |  |  |  |  |  |  |  |  |  |
| Petchaburi (*G. gallus spadiceus*) | 0.090^**^ | 0.110^**^ | 0.131^**^ | 0.145^**^ | 0.288^**^ | 0.093^**^ | 0.086 | 0.211 | 0.244 | 0.165 | 0.173 | 0.175 | 0.172 | 0.169 | 0.047 | 0.110 | 0.142 | 0.060 | 0.108 | 0.130 | 0.000 |  |  |  |  |  |  |  |  |  |  |  |  |  |  |
| Huai Yang Pan (*G. gallus spadiceus*) | 0.177^**^ | 0.193^**^ | 0.227^**^ | 0.213^**^ | 0.351^**^ | 0.153^**^ | 0.151 | 0.256 | 0.259 | 0.233 | 0.213 | 0.169 | 0.181 | 0.144 | 0.109 | 0.160 | 0.215 | 0.178 | 0.179 | 0.172 | 0.111 | 0.000 |  |  |  |  |  |  |  |  |  |  |  |  |  |
| Chiang Mai Zoo (*G. gallus spadiceus*) | 0.098^**^ | 0.086^**^ | 0.154^**^ | 0.125^**^ | 0.316^**^ | 0.053^*^ | 0.111 | 0.181 | 0.231 | 0.127 | 0.160 | 0.125 | 0.145 | 0.190 | 0.062 | 0.075 | 0.089 | 0.081 | 0.104 | 0.108 | 0.102 | 0.141 | 0.000 |  |  |  |  |  |  |  |  |  |  |  |  |
| Songkhla Zoo (*G. gallus gallus*) | 0.097^**^ | 0.117^**^ | 0.115^**^ | 0.117^**^ | 0.284^**^ | 0.034^ns^ | 0.095 | 0.161 | 0.184 | 0.165 | 0.180 | 0.165 | 0.140 | 0.177 | 0.063 | 0.076 | 0.107 | 0.102 | 0.111 | 0.134 | 0.104 | 0.150 | 0.027 | 0.000 |  |  |  |  |  |  |  |  |  |  |  |
| Songkhla Zoo (*G. gallus spadiceus*) | 0.096^**^ | 0.113^**^ | 0.121^**^ | 0.132^**^ | 0.357^**^ | 0.055^ns^ | 0.095 | 0.177 | 0.228 | 0.168 | 0.149 | 0.141 | 0.115 | 0.166 | 0.027^ns^ | 0.088 | 0.116 | 0.108 | 0.069 | 0.100 | 0.103 | 0.166 | 0.012^ns^ | -0.010^ns^ | 0.000 |  |  |  |  |  |  |  |  |  |  |
| Khon Kaen Zoo (*G. gallus gallus*) | 0.208^**^ | 0.215^**^ | 0.249^**^ | 0.226^**^ | 0.358^**^ | 0.147^**^ | 0.231 | 0.256 | 0.281 | 0.250 | 0.233 | 0.192 | 0.217 | 0.261 | 0.158 | 0.205 | 0.185 | 0.198 | 0.216 | 0.177 | 0.206 | 0.226 | 0.191 | 0.166 | 0.172 | 0.000 |  |  |  |  |  |  |  |  |  |
| Mae Hong Son | 0.179^**^ | 0.189^**^ | 0.207^**^ | 0.185^**^ | 0.322^**^ | 0.129^**^ | 0.185 | 0.244 | 0.253 | 0.235 | 0.214 | 0.189 | 0.202 | 0.212 | 0.157 | 0.165 | 0.189 | 0.176 | 0.176 | 0.167 | 0.180 | 0.210 | 0.151 | 0.145 | 0.138 | 0.190 | 0.000 |  |  |  |  |  |  |  |  |
| Chee Fah (CRRBC) | 0.187^**^ | 0.19^**^ | 0.221^**^ | 0.180^**^ | 0.362^**^ | 0.110^**^ | 0.222 | 0.225 | 0.253 | 0.214 | 0.212 | 0.178 | 0.216 | 0.225 | 0.165 | 0.180 | 0.147 | 0.165 | 0.176 | 0.165 | 0.200 | 0.217 | 0.157 | 0.159 | 0.138 | 0.179 | 0.182 | 0.000 |  |  |  |  |  |  |  |
| Fah Luang (CRRBC) | 0.188^**^ | 0.175^**^ | 0.216^**^ | 0.183^**^ | 0.391^**^ | 0.114^**^ | 0.213 | 0.236 | 0.262 | 0.230 | 0.223 | 0.187 | 0.221 | 0.230 | 0.165 | 0.176 | 0.164 | 0.154 | 0.190 | 0.191 | 0.196 | 0.217 | 0.158 | 0.163 | 0.147 | 0.193 | 0.188 | 0.050 | 0.000 |  |  |  |  |  |  |
| Fah Luang (MLRBC) | 0.296^**^ | 0.280^**^ | 0.335^**^ | 0.311^**^ | 0.468^**^ | 0.243^**^ | 0.310 | 0.353 | 0.362 | 0.338 | 0.321 | 0.277 | 0.297 | 0.328 | 0.253 | 0.264 | 0.311 | 0.267 | 0.266 | 0.259 | 0.293 | 0.312 | 0.247 | 0.240 | 0.246 | 0.275 | 0.202 | 0.219 | 0.212 | 0.000 |  |  |  |  |  |
| Chee Fah (MLRBC) | 0.275^**^ | 0.269^**^ | 0.316^**^ | 0.295^**^ | 0.463^**^ | 0.245^**^ | 0.279 | 0.351 | 0.352 | 0.333 | 0.316 | 0.268 | 0.280 | 0.315 | 0.233 | 0.253 | 0.314 | 0.256 | 0.263 | 0.253 | 0.292 | 0.329 | 0.250 | 0.224 | 0.226 | 0.281 | 0.170 | 0.238 | 0.212 | 0.137 | 0.000 |  |  |  |  |
| Dong Tao (Udonthani) | 0.191^**^ | 0.180^**^ | 0.253^**^ | 0.227^**^ | 0.385^**^ | 0.167^**^ | 0.209 | 0.292 | 0.352 | 0.264 | 0.251 | 0.208 | 0.240 | 0.275 | 0.140 | 0.242 | 0.221 | 0.168 | 0.197 | 0.204 | 0.192 | 0.251 | 0.170 | 0.186 | 0.170 | 0.217 | 0.153 | 0.220 | 0.218 | 0.227 | 0.206 | 0.000 |  |  |  |
| Wenchang (Udonthani) | 0.208^**^ | 0.202^**^ | 0.252^**^ | 0.226^**^ | 0.392^**^ | 0.158^**^ | 0.222 | 0.284 | 0.298 | 0.264 | 0.248 | 0.199 | 0.241 | 0.277 | 0.161 | 0.237 | 0.212 | 0.177 | 0.215 | 0.195 | 0.225 | 0.270 | 0.186 | 0.178 | 0.173 | 0.203 | 0.152 | 0.208 | 0.202 | 0.256 | 0.201 | 0.083 | 0.000 |  |  |
| Myanmar Fighting Chicken (Lamphun) | 0.244^**^ | 0.228^**^ | 0.330^**^ | 0.311^ns^ | 0.421^**^ | 0.220^ns^ | 0.265 | 0.300 | 0.307 | 0.239^ns^ | 0.252 | 0.202 | 0.240 | 0.303 | 0.174 | 0.198 | 0.243 | 0.188 | 0.216 | 0.195 | 0.225 | 0.292 | 0.186 | 0.165 | 0.162^ns^ | 0.202 | 0.141^ns^ | 0.178^ns^ | 0.182^ns^ | 0.207 | 0.196 | 0.134 | 0.099 | 0.000 |  |
| Lao Pa Koi (Lamphun) | 0.161^**^ | 0.164^**^ | 0.191^**^ | 0.166^**^ | 0.359^**^ | 0.112^**^ | 0.172 | 0.246 | 0.282 | 0.234 | 0.213 | 0.199 | 0.184 | 0.209 | 0.146 | 0.197 | 0.175 | 0.157 | 0.184 | 0.166 | 0.187 | 0.224 | 0.150 | 0.138 | 0.130 | 0.179 | 0.116 | 0.178 | 0.164 | 0.167 | 0.159 | 0.075 | 0.078 | 0.049 | 0.000 |

* *p* < 0.05

** *p* < 0.01

ns = not significant

References

Hata A, Nunome M, Suwanasopee T, Duengkae P, Chaiwatana S, Chamchumroon W, et al. Origin and evolutionary history of domestic chickens inferred from a large population study of Thai red junglefowl and indigenous chickens. Sci Rep. 2021; 11:2035. https://doi.org/10.1038/s41598-021-81589-7

Singchat W, Chaiyes A, Wongloet W, Ariyaraphong N, Jaisamut K, Panthum T, et al. Red junglefowl resource management guide: bioresource reintroduction for sustainable food security in Thailand. Sustainability 2022; 14:7895. https://doi.org/10.3390/su14137895

Budi T, Singchat W, Tanglertpaibul N, Wongloet W, Chaiyes A, Ariyaraphong N, et al. Thai local chicken breeds, Chee Fah and Fah Luang, originated from Chinese black-boned chicken with introgression of red junglefowl and domestic chicken breeds. Sustainability 2023; 15:6878. https://doi.org/10.3390/su15086878

Wongloet W, Singchat W, Chaiyes A, Ali H, Piangporntip S, Ariyaraphong N, et al. Environmental and socio–cultural factors impacting the unique gene pool pattern of Mae Hong-Son chicken. Animals 2023; 13:1949. https://doi.org/10.3390/ani13121949
