# Supplementary material for: Genetic admixture and diversity in Thai domestic chickens revealed through analysis of Lao Pa Koi fighting cocks
Source: PLoS One. 2023 Oct 4;18(10):e0289983. doi: 10.1371/journal.pone.0289983 (PMC10550135; doi:10.1371/journal.pone.0289983)
Supplement: S9 Table — (DOCX) [file pone.0289983.s014.docx]

**S9 Table.** List of indigenous chicken breeds and red junglefowl populations examined in the present study.

| Category | Breed/population | Location of sampling | No. of individuals examined | Refences |
| --- | --- | --- | --- | --- |
| Indigenous chicken breed | Lueng-hang-khao | Phitsanulok | 17 | Hata et al. 2021 |
|  | Chee | Phitsanulok | 10 |  |
|  | Pradu-hang-dam | Phitsanulok | 10 |  |
|  | Kheaw-Paree | Phitsanulok | 10 |  |
|  | Betong | Lopburi | 30 |  |
|  | Decoy | Phitsanulok, Sukhothai, ChaingMai | 6 |  |
|  | Fighting chicken | Bangken | 30 |  |
|  | Nin Kaset (white) | Lopburi | 10 |  |
|  | Nin Kaset (black) | Lopburi | 10 |  |
|  | Dong-Tao | Lopburi | 5 |  |
|  | Mae Hong Son | Mae Hong Son | 50 | Wongloet et al. 2023 |
|  | Chee Fah | Chaing Rai | 10 | Budi et al. 2023 |
|  | Chee Fah | Mae Hong Son | 10 |  |
|  | Fah Luang | Chaing Rai | 9 |  |
|  | Fah Luang | Mae Hong Son | 10 |  |
|  | Wenchang | Udon Thani | 13 | This study |
|  | Myanmar fighting cock | Lamphun | 2 |  |
|  | Dong-Tao | Udon Thani | 20 |  |
|  | Lao Pa Koi | Lamphun | 20 |  |
| Red junglefowl | Sa Kaeo (*G. gallus gullus*) | Sa Kaeo | 30 | Hata et al. 2021 |
|  | Chanthaburi (*G. gallus gullus*) | Chanthaburi | 30 |  |
|  | Si Sa Ket (*G. gallus gullus*) | Si Sa Ket | 30 |  |
|  | Roi Et (*G. gallus gullus*) | Roi Et | 30 |  |
|  | Khok Mai Rua (*G. gallus gallus*) | Khok Mai Rua | 30 |  |
|  | Chaing Rai (*G. gallus gallus*) | Chaing Rai | 9 |  |
|  | Huai Sai (*G. gallus gullus*) | Huai Sai | 4 |  |
|  | Huai Sai (*G. gallus spadiceus*) | Huai Sai | 15 |  |
|  | Khao Kho  (*G. gallus spadiceus*) | Khao Kho | 30 |  |
|  | Chaiyaphum  (*G. gallus spadiceus*) | Chaiyaphum | 30 |  |
|  | Petchaburi  (*G. gallus. spadiceus*) | Petchaburi | 30 |  |
|  | Huai Yang Pan  (*G. gallus spadiceus*) | Huai Yang Pan | 30 |  |
|  | Chiang Mai Zoo  (*G. gallus spadiceus*) | Chiang Mai | 7 | Singchat et al. 2022 |
|  | Songkhla Zoo  (*G. gallus spadiceus*) | Songkhla | 12 |  |
|  | Songkhla Zoo  (*G. gallus gallus*) | Songkhla | 4 |  |
|  | Khon Kaen Zoo  (*G. gallus gallus*) | Khon Kaen | 19 |  |

References

1. Hata A, Nunome M, Suwanasopee T, Duengkae P, Chaiwatana S, Chamchumroon W, et al. Origin and evolutionary history of domestic chickens inferred from a large population study of Thai red junglefowl and indigenous chickens. Sci Rep. 2021; 11:2035. https://doi.org/10.1038/s41598-021-81589-7
2. Singchat W, Chaiyes A, Wongloet W, Ariyaraphong N, Jaisamut K, Panthum T, et al. Red junglefowl resource management guide: bioresource reintroduction for sustainable food security in Thailand. Sustainability 2022; 14:7895. https://doi.org/10.3390/su14137895
3. Budi T, Singchat W, Tanglertpaibul N, Wongloet W, Chaiyes A, Ariyaraphong N, et al. Thai local chicken breeds, Chee Fah and Fah Luang, originated from Chinese black-boned chicken with introgression of red junglefowl and domestic chicken breeds. Sustainability 2023; 15:6878. https://doi.org/10.3390/su15086878
4. Wongloet W, Singchat W, Chaiyes A, Ali H, Piangporntip S, Ariyaraphong N, et al. Environmental and socio–cultural factors impacting the unique gene pool pattern of Mae Hong-Son chicken. Animals 2023; 13:1949. https://doi.org/10.3390/ani13121949
