# Supplementary material for: Genetic admixture and diversity in Thai domestic chickens revealed through analysis of Lao Pa Koi fighting cocks
Source: PLoS One. 2023 Oct 4;18(10):e0289983. doi: 10.1371/journal.pone.0289983 (PMC10550135; doi:10.1371/journal.pone.0289983)
Supplement: S1 File — (DOCX) [file pone.0289983.s015.docx]

***Supplementary Material***

**Materials and Methods**

**Mitochondrial DNA D-loop sequence analysis**

The BLASTn and BLASTx programs (http://blast.ncbi.nlm.nih.gov/Blast.cgi, accessed on 12 Jan 2023) were used to search the National Center for the Biotechnology Information database to confirm the identity of the amplified DNA fragments. Multiple sequence alignments were performed for 590 sequences in the mt D-loop dataset of The Siam Chicken Bioresource Project and Lao Pa Koi chickens using the MAFFT algorithm included in the Geneious prime version 2023.0.1 (Biomatters Ltd., Auckland, NZ) software [1]. All unalignable and any sites containing gaps and sequence primers were carefully removed and trimmed from the datasets. Estimates of the haplotype (*h*) and nucleotide (π) diversity, number of haplotypes (H), the estimator theta (*S*), overall haplotype and average number of nucleotide differences (*k*) were calculated using the mt D-loop sequences using DnaSP version 6.12.03 [2]. A statistical parsimony network of the consensus sequences was constructed using the Templeton, Crandall and Sing (TCS) algorithm in PopART version 1.7 to examine haplotype grouping and population dynamics [3]. The phylogenetic analysis was performed using Bayesian inference with MrBayes version 3.2.6 [4]. The best-fit model of DNA substitution was determined for each genetic region using Kakusan4 [5]. The Markov chain Monte Carlo (MCMC) process ran four chains simultaneously for one million generations. Following stabilization of the log-likelihood value, a sampling procedure was performed every 100 generations to obtain 10000 trees and generate a majority-rule consensus tree with mean branch lengths. All initial sample points were discarded during the burn-in period. The Bayesian posterior probability of the sampled tree population was calculated as a percentage.

**Microsatellite genotyping and data analysis**

Twenty-eight microsatellite primer sets were selected from the 30 markers recommended for chicken biodiversity studies by the Food and Agriculture Organization [6] (S2 Table). The 5՛-end of the forward primer of each primer set was labeled with a fluorescent dye (6-FAM or HEX; Macrogen Inc., Seoul, Korea). The PCR protocol was as follows: initial denaturation at 95 °C for 10 min, followed by 35 cycles of 95 °C for 30 s, 55–58 °C for 30 s, and 72 °C for 30 s, with a final extension at 72 °C for 5 min (S2 Table). The PCR products were detected by electrophoresis in 1% agarose gel. A minimum of three PCR amplifications were performed for each sample to reduce the influence of false alleles. The absence of PCR products was examined using 1% agarose gel electrophoresis. Fluorescent DNA fragment length analysis was performed using an ABI 3730XL automatic sequencer (Applied Biosystems, Foster City, CA, USA) at the DNA sequencing service of Macrogen Inc. Allelic size was determined using the Peak Scanner version 1.0 software (Applied Biosystems). Genotypic data can be accessed from the Dryad Digital Repository Dataset, https://datadryad.org/stash/share/U8_4NYOZBF8HK4UznX4HDQl-mreFCpGZ3NNMkqhoVnc, accessed on 12 Jan 2023 (S9 Table). The microsatellite DNA analysis followed the same procedure outlined in our previous animal research projects [7, 8]. The allelic frequency, number of alleles (*N*_a_), number of effective alleles (*N*_ea_), observed heterozygosity (*H*_o_), expected heterozygosity (*H*_e_) and linkage equilibrium were calculated using Arlequin version 3.5.2.2 [9]. Due to limited population sizes, deviations from the Hardy-Weinberg equilibrium were evaluated at each locus by the Markov chain Monte Carlo (MCMC) approximation of Fisher’s exact test using the “genepop” function in the package “stats” of R version 4.1.2 [10–12]. Allelic richness (*AR*) was calculated by FSTAT version 2.9.4 [13]. MicroChecker version 2.2.3 identified the null allelic markers [14]. The polymorphic information content (*PIC*) was estimated using the Excel Microsatellite Toolkit [15] and calculated for each locus. Shannon’s information index (*I*) and the fixation index (*F*) were calculated for each locus of the population using GenAlEx version 6.5 [16].

To consider the possibility of sibling-sibling or parent-offspring pairs in the captive population, we determined whether the LPK chickens were more related than random unrelated individuals. Relatedness values (*r*) were calculated for all female-female, male-male, and male-female pairs. Mean pairwise r values based on population allelic frequencies in captivity were calculated using GenAlEx version 6.5 [16]. Individual and overall *F*_IS_ with 95% confidence intervals were calculated using the LynchRt estimator [17], as implemented in the program COANCESTRY [18]. Examinations of *r* values and *F*_IS_ were conducted assuming that the averages did not differ significantly from random assortments of unrelated individuals. Pairwise genetic distances among populations were calculated based on the infinite allele model (IAM) using *F*_ST_ with corrected *p*-values in Arlequin version 3.5.2.2. and the stepwise mutation model (SMM) using *R*_ST_ in FSTAT version 2.9.4 [13]. Principal coordinate analysis (PCoA) was performed using GenAlEx version 6.5 [16] to assess the overall relationship among individuals in the population. Analysis of principal components (DAPC) was performed using the package ADEGENET 2.0 [19] in R version 4.1.2 [12]. DAPC makes no assumptions about population models [19]. It defines synthetic variables where genetic variation is maximized between clusters of individuals (*K*) and minimized within clusters. The model-based clustering method implemented in STRUCTURE version 2.3.4 was parallel run using *Structure_threader* to determine population structure [20, 21]. The run length was set to 1,000,000 MCMC replicates after a burn-in period of 1,000,000 generations using correlated allelic frequencies under a straight admixture model. The number of clusters (*K*) varied from 1 to 25, with 15 replicates for each value of *K*. The most probable number of bunches was dictated by plotting the log-likelihood of the information (ln Pr (X|*K*)) [20] over the scope of tested *K* esteems before choosing the *K* esteem value at which ln Pr (X|*K*) settled. The Δ*K* strategy was also applied using Structure Harvester [22].

To detect selection signals, phasing was performed using genotype data for each population. Molecular genetic diversity was estimated using expected heterozygosity (*H*_e_), inbreeding coefficient (*F*_IS_) and *H*_e_ of each individual/population were plotted for each microsatellite locus (total 28 loci) to predict the signature of a selective sweep. A pattern of higher *F*_IS_ coupled with low *H*_e_ was considered a signature of a selective sweep or purifying selection, whereas a low *F*_IS_ coupled with high *H*_e_ indicated the tendency of neutral or balance selection [23].

**References.**

1. Katoh K, Standley DM. MAFFT multiple sequence alignment software version 7: improvements in performance and usability. Mol Biol Evol. 2013; 30: 772–780. https://doi.org/10.1093/molbev/mst010
2. Rozas J, Ferrer-Mata A, Sánchez-DelBarrio JC, Guirao-Rico S, Librado P, Ramos-Onsins SE, et al. DnaSP 6: DNA sequence polymorphism analysis of large data sets. Mol Biol Evol. 2017; 34:3299–3302. https://doi.org/10.1093/molbev/msx248
3. Clement M, Snell Q, Walker P, Posada D, Crandall K. TCS: estimating gene genealogies. In Proceedings of the 16th International Parallel and Distributed Processing Symposium; 2002. pp.184
4. Huelsenbeck JP, Ronquist F. MRBAYES: Bayesian inference of phylogenetic trees. Bioinformatics 2001; 17:754–755. https://doi.org/10.1093/bioinformatics/17.8.754
5. Tanabe AS. Kakusan4 and Aminosan: two programs for comparing nonpartitioned, proportional and separate models for combined molecular phylogenetic analyses of multilocus sequence data. Mol Ecol Resour. 2011; 11:914–921. https://doi.org/ 10.1111/j.1755-0998.2011.03021.x
6. FAO. Molecular genetic characterization of animal genetic resources. Rome, Italy: Food and Agriculture Organization; 2011.
7. Hata A, Nunome M, Suwanasopee T, Duengkae P, Chaiwatana S, Chamchumroon W, et al. Origin and evolutionary history of domestic chickens inferred from a large population study of Thai red junglefowl and indigenous chickens. Sci Rep. 2021; 11:2035. https://doi.org/10.1038/s41598-021-81589-7
8. Singchat W, Chaiyes A, Wongloet W, Ariyaraphong N, Jaisamut K, Panthum T, et al. Red junglefowl resource management guide: bioresource reintroduction for sustainable food security in Thailand. Sustainability 2022; 14:7895. https://doi.org/10.3390/su14137895
9. Excoffier L, Lischer HE. Arlequin suite ver 3.5: a new series of programs to perform population genetics analyses under Linux and Windows. Mol Ecol Resour. 2010; 10:564–567. https://doi.org/10.1111/j.1755-0998.2010.02847.x
10. Guo SW, Thompson EA. Performing the exact test of Hardy-Weinberg proportion for multiple alleles. Biometrics 1992; 48:361–372. https://doi.org/10.2307/2532296
11. Raymond M, Rousset F. An exact test for population differentiation. Evolution 1995; 46:1280–1283. https://doi.org/10.2307/2410454
12. R Core Team. R: A language and environment for statistical computing; R Foundation for Statistical Computing. Austria, Vienna. 2023.
13. Goudet J. FSTAT (ver. 2.9.4):a program to estimate and test population genetics parameters 2003. Updated from Goudet [1995], [http://www.unil.ch/izea/softwares/fstat.html/]
14. Van OC, Hutchinson WF, Wills DP, Shipley P. MICRO‐CHECKER: software for identifying and correcting genotyping errors in microsatellite data. Mol Ecol Notes. 2004; 4:535–538. https://doi.org/10.1111/j.1471-8286.2004.00684.x
15. Park, SDE. The Excel Microsatellite Toolkit (version 3.1). Ireland: Animal Genomics Laboratory, University College. 2001.
16. Peakall RO, Smouse PE. GENALEX 6: genetic analysis in Excel. Population genetic software for teaching and research. Mol Ecol Notes. 2006; 6:288–295. https://doi.org/10.1111/j.1471-8286.2005.01155.x
17. Lynch M, Ritland K. Estimation of pairwise relatedness with molecular markers. Genetics 1999; 152:1753–1766. https://doi.org/10.1093/genetics/152.4.1753
18. Wang J. COANCESTRY: a program for simulating, estimating and analysing relatedness and inbreeding coefficients. Mol Ecol Resour. 2011; 11:141–145. https://doi.org/10.1111/j.1755-0998.2010.02885.x
19. Jombart T. adegenet: a R package for the multivariate analysis of genetic markers. Bioinformatics 2008; 24:1403–1405. https://doi.org/10.1093/bioinformatics/btn129
20. Pritchard JK, Stephens M, Donnelly P. Inference of population structure using multilocus genotype data. Genetics 2000; 155:945–959. https://doi.org/10.1093/genetics/155.2.945
21. Pina‐Martins F, Silva DN, Fino J, Paulo OS. *Structure_threader*: an improved method for automation and parallelization of programs structure, fastStructure and MavericK on multicore CPU systems. Mol Ecol Resour. 2017; 17:e268–e274. https://doi.org/10.1111/1755-0998.12702
22. Earl DA, VonHoldt BM. STRUCTURE HARVESTER: a website and program for visualizing STRUCTURE output and implementing the Evanno method. Conserv Genet Resour. 2012; 4:359–361. https://doi.org/10.1007/s12686-011-9548-7
23. Reddy UK, Abburi L, Abburi VL, Saminathan T, Cantrell R, Vajja VG, et al. A genome-wide scan of selective sweeps and association mapping of fruit traits using microsatellite markers in watermelon. J Hered. 2015; 106:166–176. https://doi.org/10.1093/jhered/esu077
